# A Chimeric Virus Approach Reveals the Matrix (M) Gene as a Critical Modulator of Mumps Virus Neurovirulence

**DOI:** 10.3390/vaccines14090774

**Published:** 2026-09-03

**Authors:** Christian J. Sauder, Malen Link, Laurie Ngo, Cheryl Zhang, Chao-Kai Chou, Wells W. Wu, Tatiana Zagorodnyaya, Majid Laassri, Steven Rubin

**Affiliations:** 1 Division of Viral Products, Office of Vaccine Research and Review, Center for Biologics Evaluation and Research (CBER), Food and Drug Administration (FDA), Silver Spring, MD 20993, USA; malenlink@gmail.com (M.L.); laurie.s.ngo@gmail.com (L.N.); tatiana.zagorodnyaya@fda.hhs.gov (T.Z.); majid.laassri@fda.hhs.gov (M.L.); steven.rubin01@gmail.com (S.R.); 2 Facility for Biotechnology Resources, Center for Biologics Evaluation and Research (CBER), Food and Drug Administration (FDA), Silver Spring, MD 20993, USA; chao-kai.chou@fda.hhs.gov (C.-K.C.); wells.wu@fda.hhs.gov (W.W.W.)

**Keywords:** mumps virus vaccine, neurovirulence, live attenuated vaccine, viral attenuation, vaccine safety, chimeric virus, genetic basis of attenuation, matrix gene, rational vaccine design

## Abstract

**Background/Objectives**: Live attenuated mumps virus (MuV) vaccine strains have significantly reduced disease incidence since their introduction in the 1960s; however, a recent resurgence of outbreaks has led to calls for the development of new vaccines to overcome what appears to be reduced effectiveness linked to waning immunity and the emergence of strains antigenically mismatched to vaccine strains. A major obstacle to the development of newer live, attenuated MuV vaccines is ensuring their safety, particularly given the virus’s neurotropic properties. Indeed, several vaccine strains licensed for use outside the US have proven to be insufficiently attenuated (such as the Urabe AM9 vaccine strain) and have caused aseptic meningitis in recipients, despite efforts to test these strains for neurotoxicity pre-licensure. Historically, attenuation was achieved empirically, and pre-clinical testing for neurovirulence safety has proven unreliable. Despite efforts in recent years, the genetic basis of attenuation of MuV vaccines remains inadequately understood. The objective of this study was to elucidate the genetic basis of neurovirulence of the Urabe AM9 vaccine strain. **Methods**: To this end, we generated a series of chimeric viruses in which genes of the highly attenuated Jeryl Lynn (JL) vaccine strain were exchanged with corresponding genes from the Urabe AM9 vaccine strain. The resulting chimeric viruses were tested for neurovirulence in a rat model and characterized for replication in vitro and in vivo. Using a multi-tiered approach consisting of independent rescue and analysis of two to three viruses per cDNA construct, possible off-target effects of identified single-nucleotide heterogeneities were mitigated. **Results**: All viruses were shown to be replication-competent in Vero cells, but differences in replication efficiencies and virus-induced neurotoxicity were observed in the in vivo model. In a rat neuronal cell line, the Urabe AM9 M and HN genes had opposite effects on the growth of chimeric JL- and Urabe AM9-based viruses. **Conclusions**: The results presented herein indicate that MuV neuroattenuation is mediated by the concerted action of multiple genes, with the matrix (M) gene exerting a dominant effect. These findings contribute to a growing body of evidence that informs the rational design of next-generation live-attenuated mumps virus vaccines.

## 1. Introduction

Mumps virus (MuV) infection can lead to a wide array of disease manifestations, including meningitis and encephalitis, reflecting the virus’s neurotropic nature [1,2]. Widespread implementation of the live, attenuated mumps vaccine starting in the late 1960s led to dramatic declines in disease incidence, with mumps becoming nearly eliminated in the United States by the early 2000s. However, a significant resurgence began in 2005, characterized by outbreaks among vaccinated young adults in high-contact settings, such as university campuses and large social gatherings [3,4,5]. These outbreaks have been hypothesized to be due to waning vaccine-induced immunity and/or antigenic mismatch between vaccine strains (predominantly genotype A) and circulating wild-type strains (primarily genotype G) [3,6,7,8,9,10]. This has prompted renewed interest in the development of more immunogenic and “updated” mumps vaccines [9,11,12,13,14,15,16,17].

All currently licensed MuV vaccines are live, attenuated viruses, and their neuroattenuation is critical to safety. However, the molecular determinants of MuV neuroattenuation remain poorly defined. MuV is a member of the family *paramyxoviridae*, genus *Rubulavirus*. The virus is enveloped and contains a non-segmented single-stranded RNA genome in negative orientation with a size of 15,384 nucleotides. The genome contains seven genes that allow expression of nine proteins: The nucleoprotein (N) is encoded by the N gene, while the nonstructural protein V (which functions as an interferon antagonist) is encoded by the P/V/I gene [18,19,20], in addition to encoding the phosphoprotein (P) and the putative nonstructural protein I, whose function remains to be determined (as detailed in [21]). Through a process involving RNA editing and co-transcriptional addition of either two or four non-templated G nucleotides at the V mRNA editing site, the transcripts for the P and I proteins are produced. The matrix (M), fusion (F), short hydrophobic (SH) and hemagglutinin/neuraminidase (HN) proteins are respectively encoded by the M, F, SH and HN genes. The matrix protein serves as a bridge connecting the viral core to the coat proteins and plays a role in viral assembly, maturation, and budding [22,23]. The HN and F proteins, which are anchored within the envelope, facilitate both attachment to and fusion with the target cell membrane. Functioning as a viroporin [24], the SH protein is believed to be situated within the envelope. Although it has been demonstrated to possess anti-apoptotic properties [25,26], it is not required for viral replication either in vitro or in vivo [27,28]. The RNA-dependent RNA polymerase (RdRp) is formed by the large (L) protein, which is encoded by the L gene, together with the P protein. The RdRp uses viral RNA that has been packaged with the N protein as a template to drive both transcription and replication of the genome. The genome is bounded at both the 3′ and 5′ termini by extragenic leader and trailer sequences, respectively, which are critical for transcription and replication. Separating the open reading frames (ORFs) of the genes are the untranslated regions that include transcriptional gene-end, intergenic and transcriptional gene-start sequences [21,29].

Due to the neurotropism of MuV, national regulatory authorities, including the FDA, require pre-clinical testing of candidate vaccines for neuroattenuation in animal models; however, this has been an empirical process since the genetic basis for attenuating the neurotoxic properties of mumps virus has not been established. This, plus the fact that animal models for neurological safety testing of MuV are not always predictive for humans [30,31], has led to clinical use of vaccines that were not sufficiently attenuated, including the widely used Urabe AM9 vaccine, which has caused aseptic meningitis in vaccinees and has subsequently been withdrawn [32,33,34,35].

**Figure 1:**
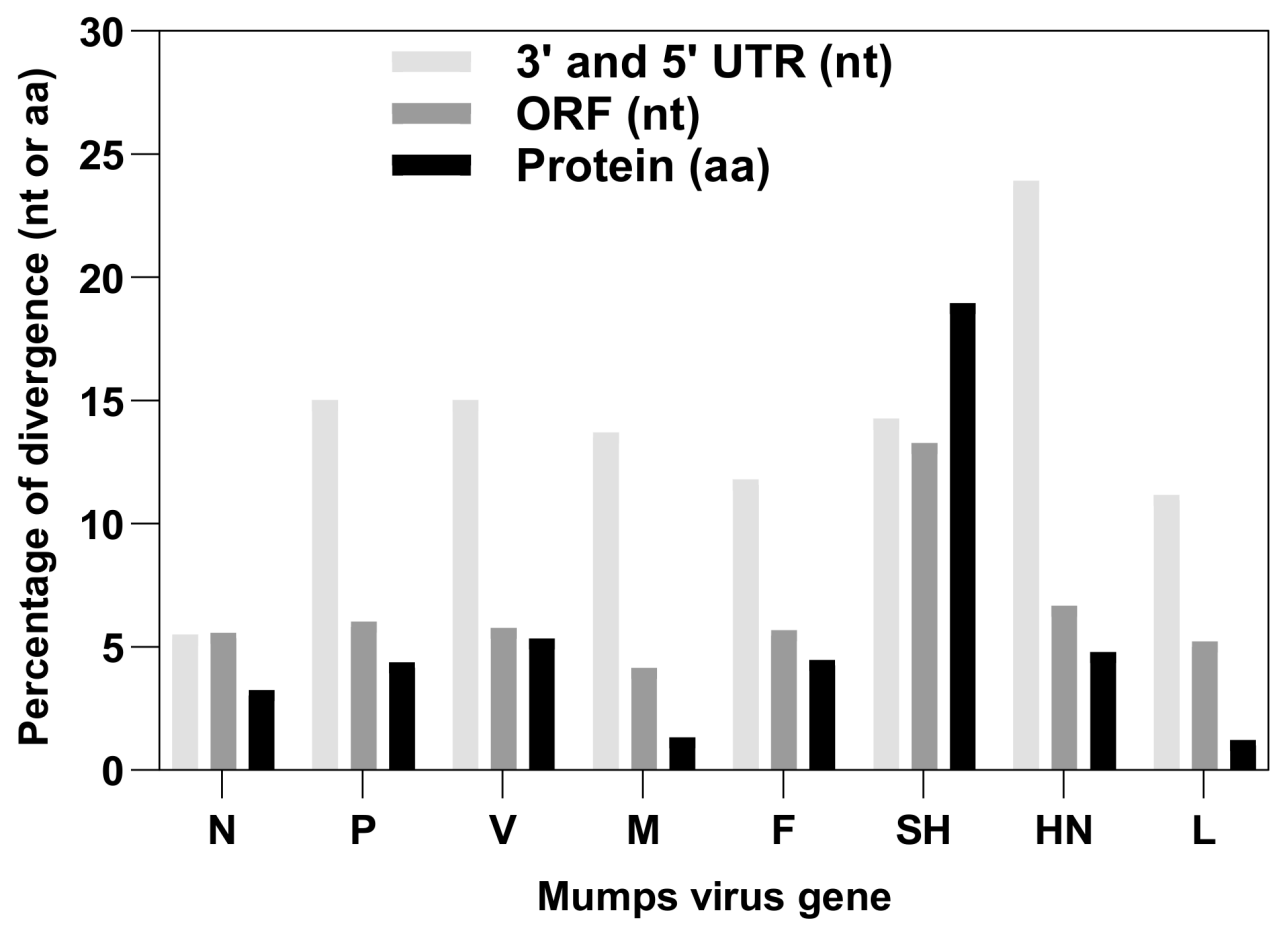
Percentage of divergence at the amino acid (aa) and nucleotide (nt) levels between the MuV vaccine strains JL and Urabe AM9. The differences in nucleotide sequences in the coding regions (ORF) and untranslated regions (3′ and 5′ UTR) of MuV genes N, P, V, M, F, SH, HN and L are shown. In addition, the percentage of divergence at the amino acid level (Protein) for the individual proteins are shown.

To support the development of safe mumps vaccines, a more reliable animal model for pre-clinical assessment of mumps neurotoxicity was developed [31,36,37], and progress has been made towards a better understanding of MuV neuroattenuation [26,28,38,39,40,41,42,43]. Notably, it was shown that inactivation of the MuV V gene confers neuroattenuation to a neurovirulent wild-type isolate [44,45]. However, the V gene in the neuroattenuated Jeryl Lynn (JL) vaccine is not inactivated, and we previously demonstrated that swapping the V gene of the neurovirulent MuV isolate 88-1961 with that of JL did not confer neuroattenuation [39]. Similarly, Lemon et al. found the mumps virus F gene to play a role in the neurovirulence of a MuV isolate adapted to grow in hamster brains [41]; however, swapping the F gene of the neurovirulent MuV isolate 88-1961 with that of JL did not confer neuroattenuation, and swapping the F gene of JL with that of the neurovirulent MuV isolate 88-1961 did not confer neurovirulence [39]. Indeed, this latter work indicated that neurovirulence is likely a multigenic trait involving several viral genes. In the present work, this issue is studied further using the insufficiently neuroattenuated Urabe AM9 (Ur) vaccine strain. The JL and Urabe AM9 MuV vaccine strains belong to genotypes A and B, respectively [46]. The differences at the nucleotide and amino acid levels between the genes and proteins of the two strains are shown in Figure 1. The SH gene and protein differ the most among all genes and proteins between the two viruses (Figure 1), consistent with the observation that the SH gene is not required for viral replication in vitro and in vivo [26,27,28]. In contrast, the M and L proteins are the most conserved proteins at the amino acid level between the two viruses, suggesting tight functional and/or structural constraints. Of note, very similar differences at the amino acid level can also be observed when comparing the JL vaccine strain with the currently circulating genotype G strains or the wildtype strain 88-1961 from genotype H.

The Urabe AM9 vaccine is of particular interest since it was shown to be a mixture of different variants, with some of them having been associated with aseptic meningitis [47]. So far, attempts to pinpoint the neurovirulence of these variants to a specific genetic marker have been inconclusive [34,38,42,48,49,50,51]. Therefore, the present study builds upon previous efforts to further explore the genetic basis for MuV neuroattenuation by generating a series of recombinant viruses consisting of combinations of genes derived from a cDNA clone of the Urabe AM9 (Ur) vaccine strain and the highly attenuated JL vaccine strain. Of the genes assessed, replacing the Ur matrix gene with that of JL had the greatest single-gene effect on decreasing neurotoxicity, with further decreases upon the incorporation of additional JL genes such as the F, SH and HN genes. Interestingly, the Ur matrix protein gene did not have a similarly influential effect on the JL strain, highlighting that the molecular basis of neurovirulence is distinct from that of neuroattenuation, which was suggested by a prior study of a different MuV strain [39]. The results presented herein, along with those from prior studies of genetic contributions to MuV neuroattenuation, are of significant relevance for the rational design of next-generation live-attenuated MuV vaccines.

## 2. Materials and Methods

### 2.1. Plasmids

**Figure 2:**
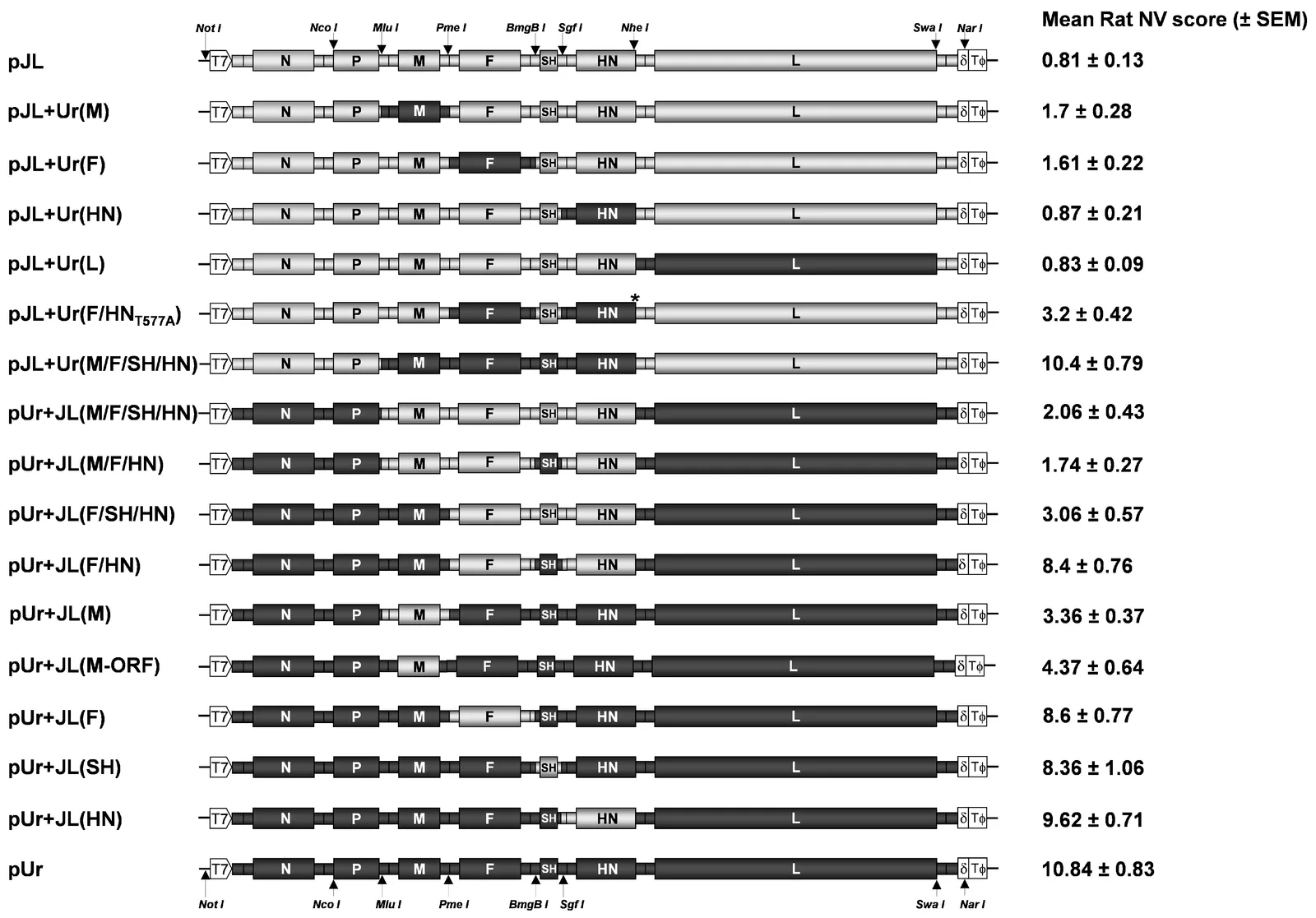
Overview of the genomic structure of all chimeric mumps viruses that were generated and subsequently evaluated in the rat neurovirulence test for the severity of virus-induced hydrocephalus as a measure of the degree of neurovirulence. All chimeric cDNA constructs as well as plasmid pUr were generated by replacing rJL-specific sequences from plasmid pJL (carrying the antigenome of the major component of the rJL vaccine strain) with those of the MuV strain Urabe AM9. Details on the construction of the recombinant plasmids are outlined in Supplemental Materials and Methods (Generation of recombinant chimeric mumps virus cDNA constructs). All boxed elements shown in gray or black denote rJL- or rUrabe-derived sequences, respectively. The locations of several restriction enzyme sites used for the generation of the various constructs are indicated. Note that the *Nhe*I site is authentic in the ORF of the rJL HN gene but is not present in that of rUr. The *Nhe*I site was introduced for cloning purposes into the rUr HN gene and has been removed in all constructs carrying the Ur HN gene except for the chimeric cDNA construct pJL + Ur(F/HN_T577A_). The presence of the *Nhe*I site in this construct is indicated with an asterisk and translates into the amino acid change T577A in the Urabe HN protein. Note that all other restriction sites indicated at the top and bottom of the figure are present in all shown constructs. In all cDNA clones, the antigenome is flanked at the 5′ end by a T7 RNA polymerase promoter and at the 3′ end by the hepatitis delta ribozyme (δ) and the T7 RNA polymerase terminator (TΦ). ORFs are indicated by large boxes. The smaller boxes between ORFs delineate untranslated regions that contain the gene-end and gene-start signals. The vertical black lines within these smaller boxes indicate the intergenic regions. The viral leader (Le) and trailer (Tr) regions are indicated. Note that genomic element sizes are for illustrative purposes and may not be to scale. Plasmid pJL is identical to pMuV(MPBS) [41]. Plasmid pUr is identical to pURABE_HN-K335_ [38]. BHK-BSR T7/5 cells were used for rescue of the viruses. These cells express T7 RNA polymerase, which binds to the T7 promoter and mediates transcription of the viral antigenome. The hepatitis delta ribozyme cleaves the nascent transcripts to generate authentic 3′ antigenomic ends. The T7 RNA polymerase also drives transcription of the N, P and L mRNAs from helper plasmids co-transfected with the respective cDNAs into BHK-BSR T7/5 cells. Once N proteins encapsulate the antigenomic RNA, the RdRp complex (assembled from the L and P proteins) binds at the antigenomic 3′ end to initiate genome transcription. The resulting viral genome serves as a template for the RdRp complex to synthesize new viral antigenomes and mRNAs encoding all viral proteins. These proteins mediate assembly, budding and release of new virions. Rescued viruses were inoculated into the brains of newborn rats as described in Materials and Methods. Mean rat neurovirulence (NV) scores (±SEM) for all rats inoculated with virus stocks rescued from the indicated chimeric recombinant viruses are shown on the right side of the panel. See Materials and Methods, as well as Results Section, for further details on neurovirulence test results for individual viruses.

The expression vectors pMuV-NP, pMuV-P, and pMuV-L, which harbor the Jeryl Lynn (JL) N, P, and L genetic sequences, respectively, were generously supplied by D. Clarke (Pearl River, NY, USA). These vectors, in conjunction with those carrying the Urabe AM9 N, P, and L polypeptides and the engineered full-length cDNA replicas for JL (pMuV(MPBS)) and Urabe AM9 (pURABE_HN-K335_), have been documented in prior investigations [38,41,52]. Vector pMuV(MPBS) (graciously contributed by P. Duprex, Center for Vaccine Research, Pittsburgh, PA, USA) is referenced hereinafter as pJL, which encompasses an *Mlu*I recognition site within the P 3′ untranslated region at nucleotide position 3171, a *Pme*I recognition site in the M 3′ untranslated region at nucleotide position 4465, a *Bmg*BI recognition site in the SH 5′ untranslated region at nucleotide position 6248, an *Sgf*I recognition site in the SH 3′ untranslated region at nucleotide position 6460, and an *Nhe*I recognition site at position 8338 in the HN open reading frame (ORF). Capitalizing on these restriction recognition sites, the M, F, SH, and HN genetic sequences of pURABE_HN-K335_ were substituted with those originating from JL separately or through diverse combinations, and conversely, to construct a battery of chimeric cDNA assemblies that were employed to generate chimeric viral particles, as illustrated in Figure 2. The insertion of the *Nhe*I recognition site at nucleotide position 8338 in the Urabe AM9 genome (via the incorporation of nucleotide substitution A8342G) results in amino acid substitution T577A in the Urabe HN polypeptide (Figure 2). This substitution was subsequently restored to A8342 (aa T577) through targeted mutagenesis in all cDNA assemblies carrying Urabe HN with the exception of vector pJL + Ur(F/HN_T577A_) (Supplemental Material and Methods-Generation of recombinant chimeric mumps virus cDNA constructs). It merits attention that the nomenclature “gene” appears consistently throughout this document, notwithstanding the circumstance that substitution of genetic sequences does not invariably encompass the complete 3′ and 5′ untranslated regions of the respective genetic sequences owing to the positioning of the restriction recognition sites employed (see Figure 2). Accordingly, in certain assemblies, untranslated regions constitute chimeric zones incorporating nucleotide sequences originating from both the JL and the Urabe AM9 isolates.

All cDNA assemblies underwent partial sequencing via Sanger sequencing methodology to validate the accuracy of the joined fragments. A comprehensive description of the assembly of the cDNA constructs utilized to generate the recombinant viral particles presented in this investigation is provided in Supplemental Material and Methods (Generation of recombinant chimeric mumps virus cDNA constructs). For purposes of clarity, hereinafter, barring explicit indication otherwise, the HN Urabe AM9 genetic sequence denotes the HN sequence that displays genomic positions A7616 and C8005 (the two positions of polymorphism formerly recognized in the Urabe AM9 vaccine [51]), specifying amino acids K335 and N464 in the HN polypeptide. Positions A7616 and C8005 are likewise present in the Urabe AM9 cDNA construct pURABE_HN-K335_ used here as the reference Urabe isolate. The vector is designated in this document as pUr.

### 2.2. Cell Lines

Vero (monkey kidney) cells (ATCC, Manassas, VA, USA; CCL-81) were propagated in Dulbecco’s modified Eagle’s medium (DMEM) or alternatively in Eagle’s minimal essential medium (EMEM), enriched with 10% fetal bovine serum (FBS). BHK-BSR-T7/5 cells [53], graciously donated by Ursula Buchholz (NIH, Bethesda, MD, USA), were expanded in DMEM supplemented with 10% FBS and 1 mg/mL Geneticin (Invitrogen, Carlsbad, CA, USA) at alternate passages. AF5 (rat neuronal) cells [54] were graciously supplied by M.V. Pletnikov (Jacobs School of Medicine and Biomedical Sciences, Buffalo, NY, USA) and were sustained in Advanced DMEM/F12 medium (Gibco/Thermo Fisher Scientific, Waltham, MA, USA) enriched with 10% FBS and L-glutamine (2 mM).

### 2.3. RT-PCR, PCR and Sanger Sequencing

All reverse transcription (RT) procedures were performed utilizing a Superscript II Reverse Transcriptase Kit (Invitrogen/Thermo Fisher Scientific, Waltham, MA, USA). PCR amplicons created for application in site-directed mutagenesis via overlap extension PCR [55] utilized Taq DNA polymerase (Promega, Fitchburg, WI, USA). For all additional PCR amplification procedures, Expand High-Fidelity polymerase (Roche Biochemicals, Burlington, NC, USA) was employed. Comprehensive information concerning the oligonucleotide primers utilized for PCR is furnished in Supplemental Material and Methods. Sanger sequencing analysis was performed using an ABI 3100 or ABI 3730xl computerized capillary nucleic acid sequencer (Applied Biosystems, Carlsbad, CA, USA), either via the CBER core laboratory or by Macrogen USA Corp (now Psomagen, Rockville, MD, USA). Sequencing results were processed with the Chromas version 1.7.6.(Technelysium, Tewantin, Australia), Jellyfish version 1.0 (Biowire.com, Mountain View, CA, USA), and Vector NTI version 11.5 (Life Technologies, Carlsbad, CA, USA) analytical software systems.

### 2.4. Viral RNA Extraction

To recover RNA from viral preparations for high-throughput sequencing (HTS), 161 µL of virus-containing cell culture supernatants underwent incubation for 2 h at 37 °C in the presence of 11,400 gel units of micrococcus nuclease (New England Biolabs, Ipswich, MA, USA). The catalytic process was halted by introducing 15 µL of 0.5 M EGTA (ethylene glycol-bis (β-aminoethyl ether)-N,N,N′,N′-tetraacetic acid; Bioworld, Thermo Fisher Scientific, Waltham, MA, USA). Viral RNA was recovered from viral particles using a QIAamp MinElute Virus spin kit (Qiagen, Germantown, MD, USA), adhering to the supplier’s recommendations, and was discharged from the column using 25 µL of DEPC-treated aqueous solution.

### 2.5. High-Throughput Sequencing (HTS)

#### 2.5.1. HiSeq

RNA obtained from rescued viral particles underwent preparation in accordance with the protocol outlined in the Illumina TruSeq Stranded mRNA Preparation Kit (Illumina, San Diego, CA, USA), excluding the polyA selection procedure. In short, roughly 100 ng of RNA underwent chemical fragmentation and conversion into complementary DNAs via reverse transcription. Double-stranded complementary DNAs underwent adenylation at the 3′ termini and received individual indices, followed by restricted-cycle (15) amplification and purification employing AMPure magnetic beads (Beckman Coulter, Brea, CA, USA). Upon evaluating the complementary DNA libraries for size and fidelity utilizing a BioAnalyser incorporating DNA 1000 reagent (Agilent Technologies, Santa Clara, CA, USA), paired-end sequencing (100 × 2 cycles) of twelve multiplexed RNA samples per lane was executed on a HiSeq^®^2500 sequencing apparatus (Illumina). Fastq documents containing HiSeq sequencing findings are made accessible at http://www.ncbi.nim.nih.gov/bioproject/1483926.

#### 2.5.2. MiSeq

RNA libraries were assembled from 100 ng of total RNA for each viral specimen (rJL + Ur(F)-1, rJL + Ur(F)-2, rJL + Ur(F)-3, rJL + Ur(HN)-1, rJL + Ur(HN)-2, rUr + JL(F/SH/HN)-1, and rUr + JL(F/SH/HN)-2) utilizing the NEBNext Ultra II RNA Library Prep Kit for Illumina (New England Biolabs, Ipswich, MA, USA). In accordance with the supplier’s protocol, RNA underwent fragmentation and priming in a single step. The fragmented RNA was subsequently reverse transcribed, and complementary strand production was completed. The resulting double-stranded complementary DNA underwent terminal restoration and coupling to Illumina paired-end linkers. Coupled products underwent size selection and purification utilizing AMPure beads (Beckman Coulter, Brea, CA, USA). The libraries were subsequently amplified through twelve cycles of PCR employing multiplexed primer sets, followed by terminal purification utilizing AMPure beads. The fidelity and size characteristics of the final nucleic acid libraries underwent assessment utilizing a 4200 TapeStation apparatus incorporating High Sensitivity D1000 ScreenTape (Agilent Technologies, Santa Clara, CA, USA). Libraries underwent quantitation utilizing a Qubit fluorometer. Contingent on concentration and mean fragment size, the libraries underwent pooling and sequencing (paired-end sequencing, 250 × 2 cycles) on a MiSeq apparatus (Illumina, San Diego, CA, USA) to generate 2 × 250 bp paired-end reads. Fastq documents containing MiSeq sequencing findings are accessible at http://www.ncbi.nim.nih.gov/bioproject/1483926.

The sequencing findings underwent analysis utilizing the specialized high-performance integrated virtual environment (HIVE) infrastructure made accessible for community utilization at George Washington University at hive.biochemistry.gwu.edu. Reads retrieved for complete-genome HTS underwent refinement through implementation of a threshold that eliminated the oligonucleotide sequences positioned at the ends of reads. Furthermore, sequencing reads underwent reduction to sustain a quality score of Q20. The two HIVE algorithms, HIVE-hexagon and HIVE-heptagon, utilized for examining single-nucleotide heterogeneities, insertions and deletions in the reads retrieved via HTS, were previously characterized [56,57].

### 2.6. Rescue of Viruses from cDNA

The BHK-BSR-T7/5 cell line was expanded to 95% saturation in 6-well compartments in the presence of 1 mg/mL Geneticin and an aggregate volume of 2 mL DMEM/9% FBS. Two hours before transfection, the medium was substituted with 2 mL of newly formulated medium devoid of geneticin. For the rescue of JL viral particles utilizing vector pJL and for the rescue of all chimeric recombinant viral particles displaying the JL N, P, and L genetic sequences, cells underwent transfection with a combination encompassing 300 ng of pMuV-NP, 50 ng of pMuV-P, 200 ng of pMuV-L, and 6.95 µg of the matching full-length cDNA constructs. For the rescue of Urabe AM9 viral particles utilizing vector pURABE_HN-K335_ and for the rescue of all chimeric recombinant viral particles displaying the Urabe AM9 N, P, and L genetic sequences, cells underwent transfection with a combination encompassing 225 ng, 37 ng, and 171 ng of the vectors transcribing the Urabe AM9 N, P, and L polypeptides, respectively, and 6.95 µg of the matching cDNA full-length constructs. Ultimately, for the rescue of recombinant JL viral particles harboring the JL N and P genetic sequences and the Urabe AM9 L genetic sequence (pJL + Ur(L)), transfection was accomplished utilizing a combination consisting of the vectors transcribing the JL N and P genetic sequences and the Urabe AM9 L genetic sequence and 6.95 µg of pJL + Ur(L). For all transfections, nucleic acid was integrated with 30 µL of Lipofectamine 2000 (Invitrogen/Thermo Fisher Scientific) in an aggregate volume of 530 µL alongside Opti-MEM I Reduced Serum Medium (Gibco/Thermo Fisher Scientific), functioning as the diluent. All cells initially underwent transfer to a 25 cm^2^ container 48 h post-transfection. Contingent on the magnitude of syncytial formations present, cells underwent subsequent transfer into four to six wells of a 6-well container or into a T75 cm^2^ container 2–4 days later. Supernatants underwent retrieval 6 to 11 days post-transfection and were clarified through centrifugal procedures at 1200 revolutions per minute for 5 min, and aliquots underwent retention at −70 °C. To formulate viral preparations, viral particles underwent expansion in Vero cells. Vero cells grown to confluent monolayers in T75 cm^2^ containers underwent exposure to 5 mL of supernatants originating from BHK-BSR-T7/5 cells and were kept in a volume of 20 mL of medium for 3–9 days. Supernatants underwent retrieval and were clarified via centrifugal procedures at 1200 revolutions per minute for 5 min, and aliquots underwent retention at −70 °C. With respect to the rescue of viral particles rUr-3, rUr + JL(HN)-3 and rUr + JL(F/HN)-3, T75 cm^2^ containers underwent freezing at −70 °C. Cells were subsequently dislodged into the medium and aggregated via centrifugal procedures at 1200 revolutions per minute. The supernatants underwent retrieval, and aliquots underwent freezing at −70 °C. Viral concentrations underwent quantification by plaque titration. Viral particles rescued from the same identical cDNAs underwent designations -1, -2, and -3, respectively, exemplified by rUr-1, rUr-2, and rUr-3.

### 2.7. In Vitro Multicycle Growth Kinetics in Vero Cells and AF5 Cells

The majority of viral replication kinetics in Vero cells was conducted in 6-well compartments. Replication kinetics for three viruses (rJL-1; rJL + Ur(F)-3; rJL + Ur(F/HN_T577A_)-1) were conducted in T75 cm^2^ containers (Table 1; Figure S1). Cells underwent seeding and expansion until saturation. Cells (2–3 × 10^6^ per compartment in a 6-well container or 1–2 × 10^7^ per T75 cm^2^ container) underwent infection at a multiplicity of infection (MOI) of 0.05. Viral preparations underwent dilution in EMEM to an aggregate volume of 500 µL per compartment (6-well container) or in DMEM to an aggregate volume of 5 mL (T75 cm^2^ container).

Following incubation for 60 min at 37 °C on a rocking apparatus, supernatants underwent displacement, and cells were washed twice with phosphate-buffered saline (PBS). Then, 2 mL (6-well compartment) or 20 mL (T75 cm^2^ container) of expansion medium (EMEM or DMEM supplemented with 10% FBS) was added. For the first time point in 6-well format, 0.4 mL to 1 mL of medium underwent displacement within 60 min post-inoculation and was replenished with the matching volume of newly formulated expansion medium. For all subsequent time points, 200 µL aliquots underwent displacement and were replenished with 200 µL of newly formulated medium. For T75 cm^2^ containers, 1–2 mL of supernatant underwent displacement per time point and were replenished with a matching volume of newly formulated expansion medium. Multicycle replication kinetics in AF5 cells (passage level 87–89) were conducted in 6-well containers, with two compartments used per time point and virus. Cells underwent expansion in expansion medium until saturation and underwent inoculation at an MOI of 0.05 (cellular concentration at the phase of contact ranged from 1.15 × 10^6^ to 1.57 × 10^6^ cells/compartment). Viral particles underwent dilution in EMEM to an aggregate inoculation volume of 0.5 mL per compartment. Subsequent to inoculation, cells underwent washing with PBS twice, and 2 mL of newly formulated expansion medium was added. For the first time point, 0.8 mL of supernatant underwent displacement either immediately following the introduction of the newly formulated medium (0 h time point) or subsequent to 1 h of inoculation (1 h time point) and was substituted with 0.8 mL of newly formulated expansion medium. For all subsequent time points, 0.4 mL underwent displacement and was replenished with 0.4 mL of newly formulated expansion medium. Samples underwent freezing at −70 °C while awaiting viral quantification through plaque titration.

### 2.8. In Vivo Growth Kinetics

Litters of Lewis/SsNHsd rats (Harlan; since 2015: Envigo (Indianapolis, IN, USA)) no older than 24 h underwent direct cerebral inoculation with 20 µL of EMEM harboring 100 plaque-forming units (pfu) of viral particles utilizing a 50 µL Hamilton syringe (Hamilton Company, Reno, NV, USA). All inoculations were positioned approximately 1 to 2 mm to the left of the longitudinal axis and equidistant from the bregma and lambda anatomical landmarks. On days 1, 2, 3, 4, 5, 6, and 8, the brains from 4–9 rats from the respective virus groups underwent sterile harvesting and were homogenized into a 20% (*w*/*v*) suspension in EMEM incorporating 2% FBS via dounce-homogenization methodology, followed by three brief bursts (10 s each) of ultrasonic treatment. Homogenized preparations underwent clarification through centrifugal separation at 2000× *g* at 4 °C for 10 min and were retained at −70 °C pending assessment of viral abundance per gram of brain tissue. For quantification of the genuine viral inoculum, an aliquot of the respective diluted viral preparations employed for inoculation underwent back-titration. Viral abundance per gram of brain tissue underwent quantification utilizing the median weight of five brain tissues extracted from 16–24-h-old rat offspring.

### 2.9. Virus Titration

Quantification of specimens from the in vivo and in vitro viral replication kinetics was performed in a 24-compartment container in duplicate. Quantification of the concentration of the rescued viruses was accomplished in a 12-compartment container in triplicate. Quantifications were performed in accordance with previously documented techniques [39,51].

### 2.10. Neurovirulence Phenotype Determination

The neurovirulence characteristics of all recombinant viruses underwent evaluation utilizing the newborn rat-based approach previously explained [31,37,38,39]. Briefly, newborn Lewis/SsNHsd rats underwent inoculation with 100 plaque-forming units (pfu) of viral particles intracranially (as clarified previously under in vivo replication kinetics), and on day 30 post-inoculation, rats underwent euthanization via CO_2_ asphyxiation. Cerebral tissues underwent retrieval, were partitioned along the midline, and were fixed in 10% neutral buffered formaldehyde at 4 °C for 4–5 days. Cerebral tissues were embedded in paraffin utilizing a tissue preparation apparatus (TISSUE-TEK, V.I.P.3000, Vacuum Infiltration Processor, Sakura Finechemical Co. Ltd., Tokyo, Japan). Two 10-µm-thick midline sections were chosen at a standardized distance from both sides of the anatomical midline, and corresponding sections were stained utilizing hematoxylin and eosin (H&E). The neurovirulence (NV) score quantifies the severity of MuV-prompted hydrocephalus and is calculated by gauging the dimensional expanse of the cerebrum (discounting the hindbrain) and that of the lateral ventricle on each tissue section utilizing Image Pro Plus version 7.0 (Media Cybernetics, Silver Spring, MD, USA) or ImageJ image analysis software version 1.52m (open-source software, NIH, Wayne Rasband). This is presented in Figure S2. The mean ratio (percentage) of these dual assessments on each of the two tissue sections per rat brain undergoes quantification, and this undergoes reiteration for a supplementary H&E-stained section (consisting of the dual brain hemispheres) of the same brain tissue block. The mean ratio (percentage) of these dual assessments on each of the two tissue sections per rat brain is the neurovirulence score for that distinct brain. In certain instances, only one brain section (containing the dual brain hemispheres) per rat underwent assessment. The neurovirulence score for each distinct recombinant virus is calculated as the mean neurovirulence score of all brains within a treatment group. At least three rat litters were utilized for a distinct viral preparation.

### 2.11. Statistical Analysis

Neurovirulence scores between different viruses, as well as between different rescues derived from the same virus preparation, were compared utilizing a test for normality, followed by the Mann–Whitney test unless otherwise indicated. The assessment, including regression analysis, was conducted utilizing GraphPad Prism software (version 10.1.) (Boston, MA, USA).

## 3. Results

### 3.1. Combined Replacement of the Urabe AM9 M, F, SH, and HN Genes with Those of JL Confers Attenuation of the Chimeric Urabe AM9 Virus

**Figure 3:**
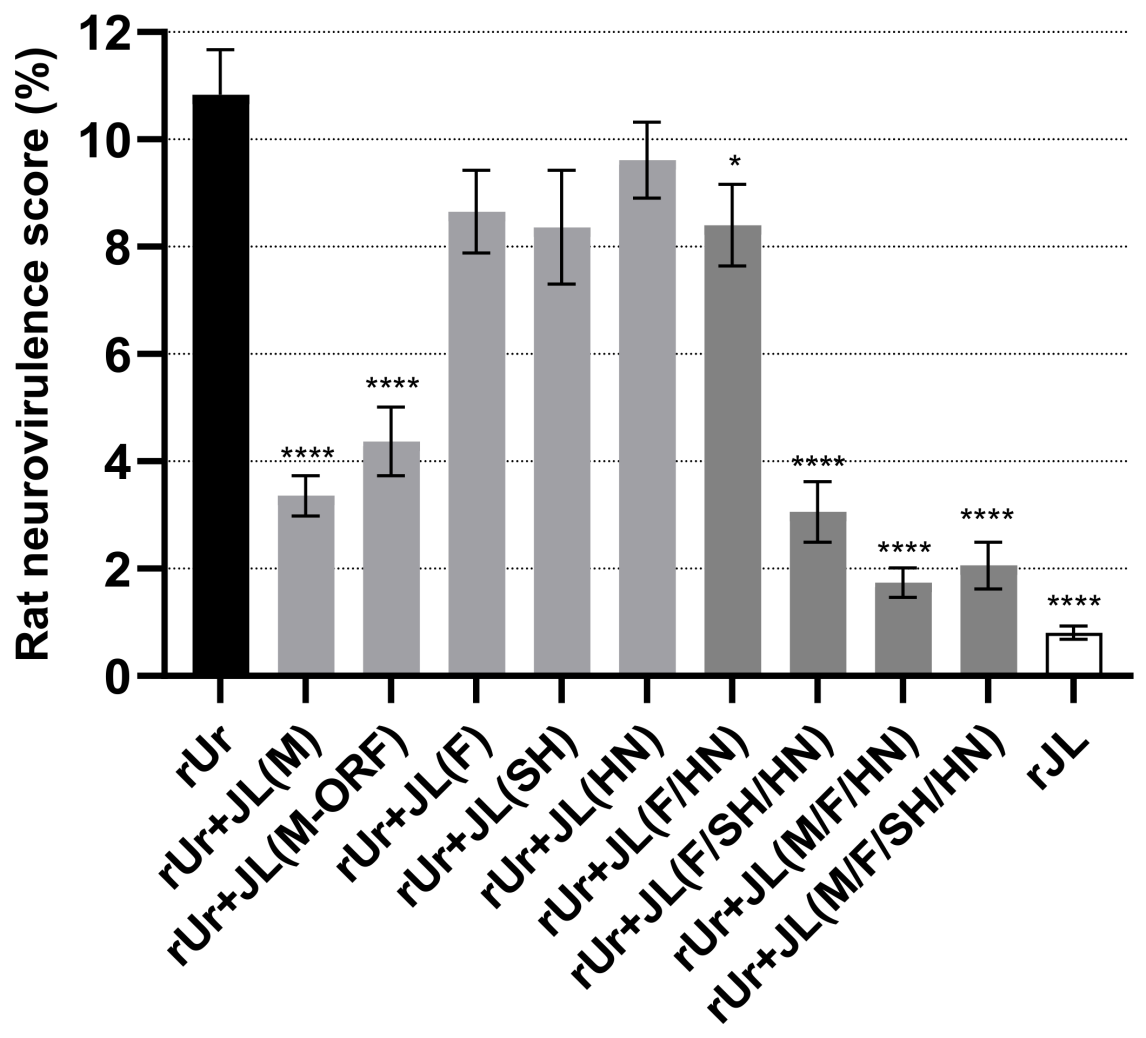
Rat NV scores of chimeric viruses in which genes of recombinant MuV vaccine strain Urabe AM9 (rUr)(black bar) were replaced individually (light grey bars), or in combination (dark grey bars), with corresponding genes from recombinant MuV vaccine strain JL (rJL) (white bar). Bars represent the mean (±SEM) NV score of all rats inoculated with virus stocks rescued from the indicated chimeric recombinant viruses (values are provided in Figure 2). The mean represents inoculation of rats with virus preparations that were obtained from two or three independent rescue events (see Table 1 and Table S2 for details on individual virus rescues and the number of animals analyzed per rescued virus; see Figure S3 for a graphical depiction of rat NV scores of individual rats per rescued virus). Statistical analysis was done using the Mann–Whitney test comparing the mean NV score of each chimeric virus with that of rUr. Note that except for the dataset for virus rUr + JL(HN), all datasets failed the test for normal distribution. (*: *p* = 0.0215; ****: *p* < 0.0001).

To identify genetic determinants of Urabe AM9 linked to neuroattenuation, we assessed the effect on neurovirulence when the M, F, SH and HN genes of Urabe AM9 were replaced individually or in combination with those from the attenuated JL strain, resulting in chimeric cDNAs termed pUr + JL(M), pUr + JL(F), pUr + JL(SH), pUr + JL(HN), pUr + JL(F/HN), pUr + JL(M/F/HN), pUr + JL(F/SH/HN), and pUr + JL(M/F/SH/HN) (Figure 2). To mitigate confounding by second-site mutations, for each cDNA construct at least two virus stocks were independently rescued and tested for their neurovirulence potential in the newborn rat assay. Results for individual virus stocks are summarized in Table 1 and results for individual rats for each virus stock are depicted in Figure S3 and Table S2. Mean NV scores were eventually calculated from all rats inoculated with virus stocks generated from the same cDNA constructs, and they are each listed in Figure 2 and graphically depicted in Figure 3.

As previously reported, the highly neuroattenuated recombinant JL virus (rJL) and the neurovirulent recombinant Urabe AM9 virus (rUr) exhibited mean NV scores of 0.81 and 10.84, respectively [38,39]. Individual replacement of the Urabe AM9 F, SH or HN genes with those from the JL strain resulted in slightly reduced NV scores compared to rUr, but the reductions were not statistically significant. In contrast, replacement of the Urabe AM9 M gene with that of JL (rUr + JL(M)) conferred a statistically and clinically significant 70% mean reduction in neurovirulence (range 45–85% across three independently rescued viruses, Figure S3). However, as depicted in Figure 2 and Figure S4, due to the initial cloning strategy, the flanking 3′ and 5′ UTRs of the M gene and the P gene 5′ UTR of pUr + JL(M) were also of JL origin. Therefore, the observed attenuation could be intrinsic to the JL M protein or due to changes in expression levels of the M gene conferred by the regulatory sequences flanking the JL M gene. To discriminate between these effects, a second construct was generated, in which only the Urabe AM9 M ORF was replaced with that of JL (pUr + JL(M-ORF), Figure 2 and Figure S4). No statistically significant difference was observed in neurovirulence between this construct and the NV score of rUr + JL(M) (*p* = 0.501, Mann–Whitney test), demonstrating that the attenuation is conferred by a qualitative change in the M protein (Figure 3) and not by differences in the regulatory elements flanking the M ORF. Replacing both the F and HN genes of Urabe AM9 with those of JL (pUr + JL(F/HN)) conferred a small but statistically significant 23% mean reduction in neurovirulence when compared to rUr (ranging from a 10% increase to a 53% reduction, Figure S3 and Table 1). A 72% mean reduction in neurovirulence was achieved with rUr + JL(F/SH/HN), and when the JL matrix gene was added as in rUr + JL(M/F/SH) and rUr + JL(M/F/SH/HN), the resulting neurovirulence was close to that of rJL (1.74 and 2.06, respectively, vs. 0.81; Figure 3 and Table 1). While there was still a statistically significant difference between the NV scores of these viruses and rJL (*p* < 0.001; Mann–Whitney test), the difference is unlikely to be of biological significance.

### 3.2. The Urabe AM9 M, F, SH and HN Genes Together Confer Neurovirulence to the Chimeric JL Virus

**Figure 4:**
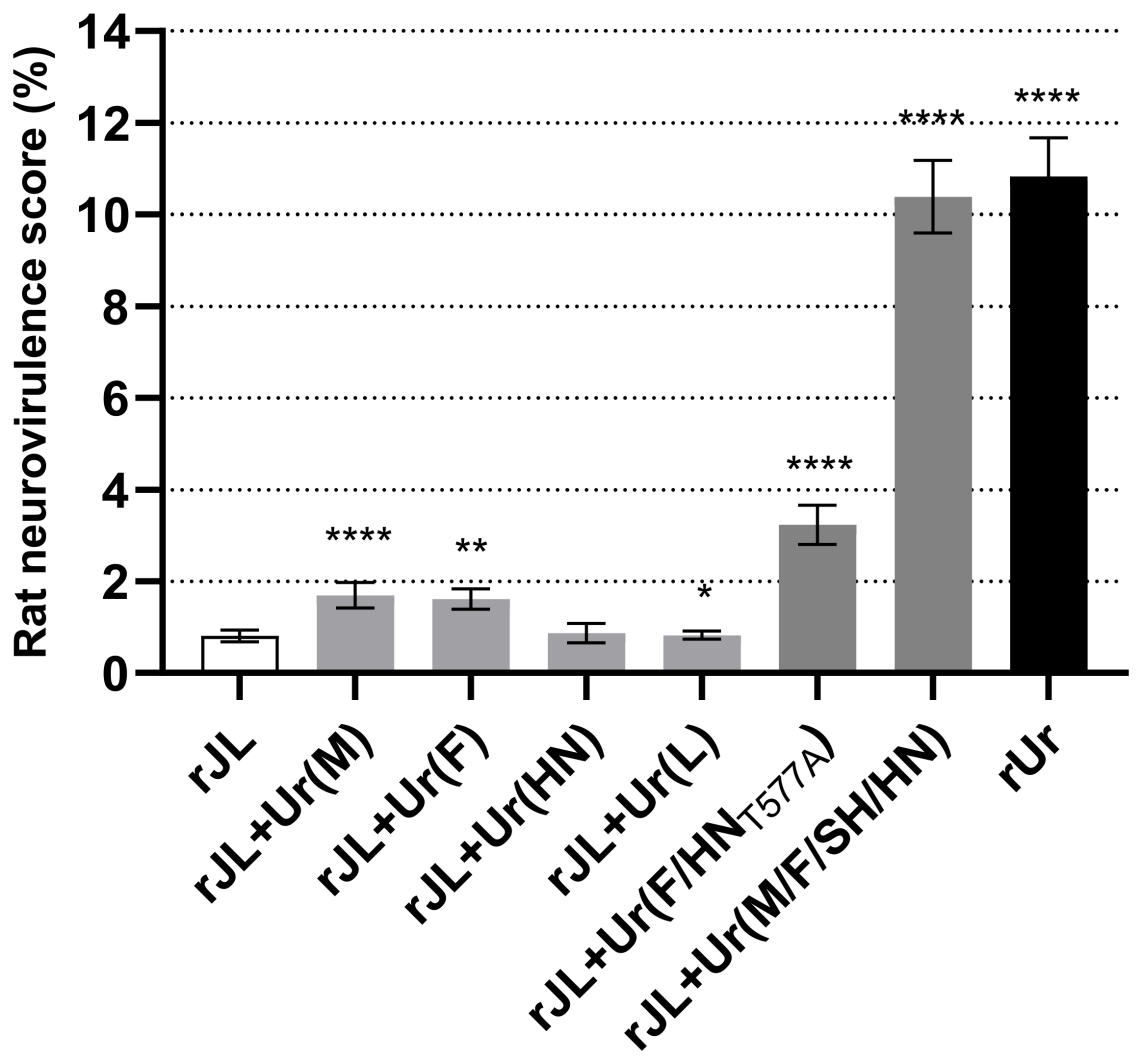
Rat NV scores of chimeric viruses in which genes of recombinant MuV vaccine strain JL (rJL)(white bar) were replaced individually (light grey bars) or in combination (dark grey bars) with corresponding genes from recombinant MuV vaccine strain Urabe AM9 (rUr, black bar). Bars represent the mean (±SEM) NV score of all rats inoculated with virus stocks rescued from the indicated chimeric recombinant viruses (mean values are provided in Figure 2). Note that except for rJL + Ur(L), the mean represents inoculation of rats with virus preparations that were obtained from at least two independent rescue events (see Table 1 and Table S3 for details on individual virus rescues and number of animals analyzed per rescued virus; see Figure S5 for a graphical depiction of RN scores of individual rats per rescued virus). Statistical analysis was done using the Mann–Whitney test, comparing the mean NV score of each chimeric virus with that of rJL. Note that except for the dataset for virus rJL + Ur(M/F/SH/HN), all datasets failed the test for normal distribution. (*: *p* = 0.0147; **: *p* = 0.0066; ****: *p* < 0.0001).

Having shown that combined replacement of the Urabe AM9 M, F, SH, and HN genes with those of JL conferred nearly complete attenuation, we asked whether, conversely, neurovirulence could be conferred to the JL virus by replacing the JL M, F, SH and HN genes with those of Urabe AM9. The resulting chimeric virus rJL + Ur(M/F/SH/HN) exhibited a degree of neurovirulence that was statistically indistinguishable from that of the parental rUr virus (*p* = 0.5044, Mann–Whitney test; Figure 2, Figure 4 and Figure S5; Table 1 and Table S3). The NV scores for individual gene replacements overall trended higher than those for rJL, but the differences were small and likely not biologically significant (Figure 2, Figure 4 and Figure S5, Table 1). An additive effect on neurovirulence was observed when the JL F and HN genes were replaced together with those of Urabe AM9 (rJL + Ur(F/HN_T577A_)), with a mean NV score approximately four-fold higher compared to that of rJL (Figure 4). Nevertheless, combined replacement of the F and HN genes was not sufficient to render the JL virus as neurovirulent as Urabe AM9 or rJL + Ur(M/F/SH/HN). To this end, we did not further examine whether additional individual replacement of the M or SH genes in rJL + Ur(F/HN_T577A_) further increased neurovirulence to the level of rUr.

Taken together, these data suggest that the presence of the Urabe AM9 M gene, in addition to the Urabe AM9 F and HN genes, and possibly the SH gene, is required to impart a wildtype neurovirulence phenotype to a chimeric virus with a JL-based backbone. Likewise, swapping the F and HN genes of Urabe AM9 with those of JL, in combination with the M and/or SH genes of JL, confers an attenuated phenotype to the recombinant virus. Notably, however, the JL M gene had a more pronounced effect on rUr than the Urabe AM9 M gene had on rJL. Additionally, the data demonstrate that neither full neurovirulence (as seen with Urabe AM9) nor complete neuroattenuation (as is the case with JL) could be conferred to either rJL or rUr, respectively, by swapping only a single gene, indicating that neurovirulence and neuroattenuation of the Urabe AM9 and JL viruses are multigenic traits, consistent with the conclusion from studies of the MuV wildtype strain 88-1961 [39].

### 3.3. Identification of Off-Target Nucleotide Heterogeneities in Recombinant Viruses

It was previously shown that recombinant MuV rescued from plasmids in BHK-BSR-T7/5 cells and propagated in Vero cells is prone to generating mutations, mainly in the form of single-nucleotide heterogeneities (SNHs), which could possibly affect the MuV neurovirulence phenotype [38,39,51,59,60]. To mitigate this possibility, two to three viruses per cDNA construct were independently rescued and tested for neurovirulence, and their quasispecies nature was interrogated using high-throughput sequencing (HTS) or conventional Sanger sequencing (Table 2 and Table S1). For the sake of simplicity, in this overview, SNHs were only considered when the substituted nucleotide represented at least 10% of the heterogeneity. Based on this threshold, 34 of the 41 recombinant viruses sequenced in this study displayed SNHs (Table 2). For 10 of these 34 viruses, the SNHs at one to three positions were ≥50%. It is noteworthy that in three of the seven viruses with only one variant at a prevalence higher than 50%, the variant was in the form of an insertion in a gene-end signal (discussed below). All other variants that exceeded the SNH threshold of 50% were single-nucleotide substitutions in the L gene (Table S1).

In addition to the SNHs, the F gene-end signal was found to be extended by an additional U in 13 viruses. However, in only two of these viruses, variants with the additional U were prevalent (73% and 60% in rUr + JL(F)-1 and rUr + JL(F/HN-1), respectively), whereas in the other viruses, the variants with the additional U were in the minority, ranging between 12% and 37% (Table S1). These findings are in agreement with our previous observation that generation and propagation of recombinant MuV can result in viruses that exhibit an additional U residue in the homopolymeric U tract (six U) of the F gene-end signal, and our published data suggest that the extension of the F gene-end signal does not have an impact on MuV neurovirulence [58,61]. All members of the family *paramyxovirinae*, such as measles virus, exhibit genomes with a polyhexameric (6n) length, and for some of these viruses, strict adherence to this “rule of six” has been shown to be essential for efficient replication. Extension of the F gene-end signal by an additional U residue violates this rule of six and creates genomes of a nonpolyhexameric length (6n + 1). It was therefore important to investigate whether MuV with genomes that are nonpolyhexameric would be able to replicate efficiently and whether this would have an impact on neurovirulence. By using recombinant viruses with a 6n + 1 genome length, we previously showed that viruses recovered from 6n + 1 cDNAs replicated efficiently and maintained a nonpolyhexameric genome length over several cell passages until eventually genomes prevailed with a 6n genome length. These data demonstrated that while polyhexameric genomes are preferred by the viral polymerase, this requirement is not strict [61].

Of note, in virus rUr + JL(F/SH/HN)-2, an insertion of an additional U residue was identified in the homopolymeric tract (6 U) in the HN gene-end signal, albeit in only 12% of the populations of variants. Moreover, 59% of the genomes of virus rUr + JL(F/HN)-3 exhibited an extension by one extra U of the homopolymeric tract (6 U) in the M gene-end signal. Nevertheless, it is unknown whether the extensions of the HN and M gene-end signals influenced neurovirulence. In summary, while an effect on neurovirulence of identified SNHs cannot be ruled out, in the majority of SNHs, the mutant variant was less prevalent compared to the authentic nucleotide, and therefore, effects on NV would be diluted by the predominant variant. Moreover, only a few of the prevalent mutations were radical aa changes. Finally, the two-to-three-tiered approach of virus rescue and analysis mitigates off-target effects on NV.

### 3.4. Assessment of Efficient Growth of Recombinant Viruses in Vero Cells

To verify efficient replication of all rescued viruses depicted in Figure 2, virus growth kinetics were assessed in Vero cells following infection at an MOI of 0.05. As shown in Table 1 and Figure S1, the vast majority of rescued viruses reached peak virus titers between two and three days post infection. Most peak virus titers ranged from 6.1 to 7.2 pfu/mL log_10_, being typical of the peak titers of the rJL and rUr viruses, respectively. However, both viruses rescued from pJL + Ur(HN) only grew to 5.15 and 4.74 log_10_ pfu/mL, respectively. We noticed that, in contrast to rJL, infection of Vero cells with these viruses (rJL + Ur(HN)-1 and 2) at an MOI of 0.05 led to a faster formation of large syncytia that consumed all cells by day 2 post infection (p.i.) and subsequently led to a more rapid lysis of cells by day 3 p.i., likely explaining the reduced titers of these viruses compared to those measured for rJL (Figure S6). This suggests that the heterologous combination of the Urabe AM9 HN protein with the JL F and JL M proteins might either affect virus binding, entry and fusion or proper budding and release of progeny viruses. Indeed, in support of this hypothesis, viruses rescued from pJL + Ur(F/HN_T577_) grew to higher titers compared to rJL + Ur(HN) (Table 1 and Figure S1). Conversely, it is noteworthy that replacing the Urabe HN gene with that of the JL HN gene in the three viruses rescued from rUr + JL(HN) conferred a replicative advantage in Vero cells, with peak titers of all three viruses exceeding those observed for rUr 1–3 (Table 1, Figure S1). It should be noted that differences in growth curves among several viruses rescued from the same cDNA plasmid were observed (Figure S1), such as rUr(F)1–3 and rUr + JL(F/HN)-1–3. It is likely that these differences are due, at least in part, to the nucleotide heterogeneities identified in the individual rescued viruses as outlined above and summarized in Table S1. Taken together, the data demonstrate that all viruses were replication-competent, with minor variations in replication efficiency observed depending on individual rescues and constellations of replaced proteins in chimeric viruses.

### 3.5. Growth Efficiency in the Newborn Rat Brain Correlates with Rat NV Scores

**Figure 5:**
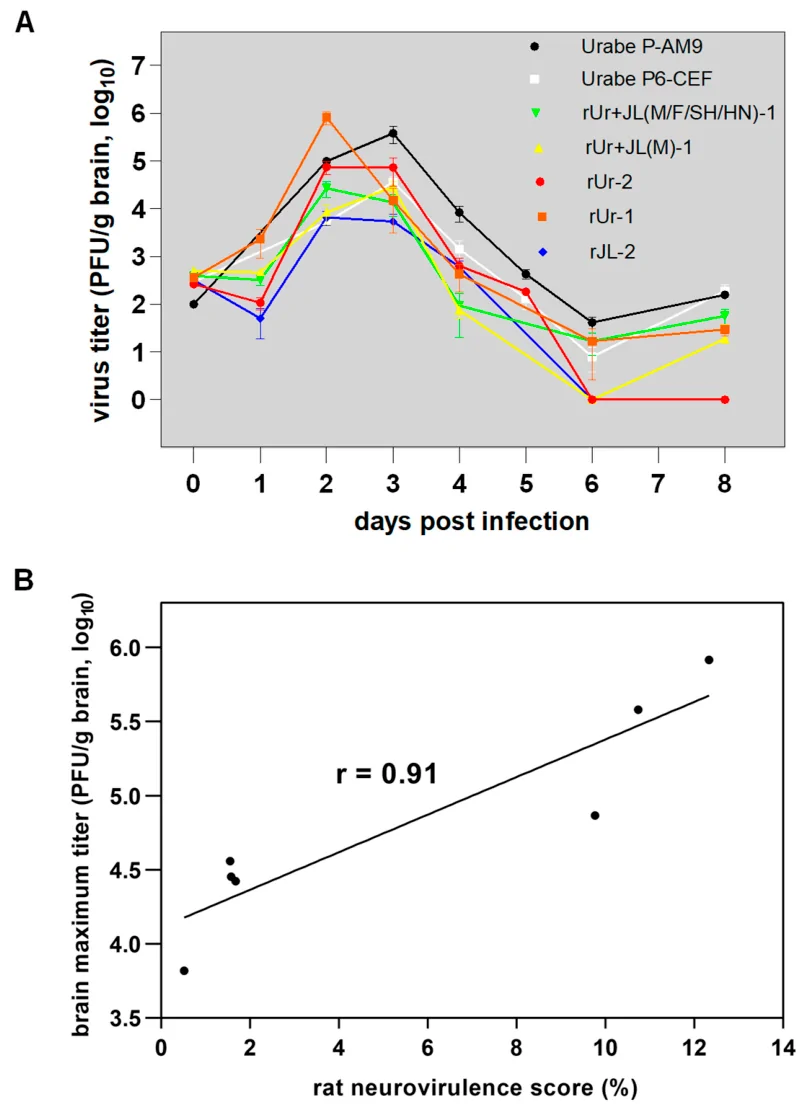
Virus growth kinetics in newborn rat brains and correlation with neurovirulence scores. (**A**): Newborn rats were inoculated intracerebrally with a selection of five recombinant viruses as well as the nonrecombinant viruses Urabe P-AM9 and Urabe P6-CEF. Data for these two nonrecombinant viruses as well as for rJL-2 are historic data ([51] and [39], respectively). Brains were collected at the indicated time points and virus titers were determined per gram of brain tissue. Shown are mean values (±SEM). (**B**): Peak virus titers in brains were plotted against NV scores for the seven viruses, and regression analysis was performed. The coefficient of correlation (r value) is indicated.

We previously demonstrated a good correlation between rat NV scores and peak virus titers in brains following neonatal intracerebral inoculation [39], and this was confirmed here. Since recombinant viruses rUr + JL(M) and rUr + JL(M/F/SH/HN) exhibited significant reductions in the rNV score, one rescued virus each was analyzed for peak brain virus titer, together with two rescued recombinant Urabe AM9 viruses and one rescued recombinant JL virus. The data were also compared with historic brain titration data for the non-recombinant Urabe P-AM9 vaccine virus (original Urabe AM9 virus passaged twice in Vero cells) and the neuroattenuated Urabe P6-CEF virus (Urabe P-AM9 virus following six serial passages in chicken embryo fibroblast (CEF) cells [51]). As shown in Figure 5A, the Urabe P-AM9 virus and both recombinant Urabe AM9 viruses (rUr-1 and rUr-2) reached the highest brain virus titers. In contrast, the recombinant JL virus (rJL-2) grew to the lowest titer. Viruses rUr + JL(M), rUr + JL(M/F/SH/HN) and Urabe P6-CEF grew to slightly higher titers than rJL-2, but they were lower than those of the Urabe viruses, in agreement with the lower rat NV scores of these viruses. Plotting the peak virus titers of these viruses against the determined rat NV scores confirmed the good correlation between these two parameters (Figure 5B, r = 0.91).

### 3.6. Correlation of Rat Neurovirulence Data with Peak Virus Titers In Vitro

**Figure 6:**
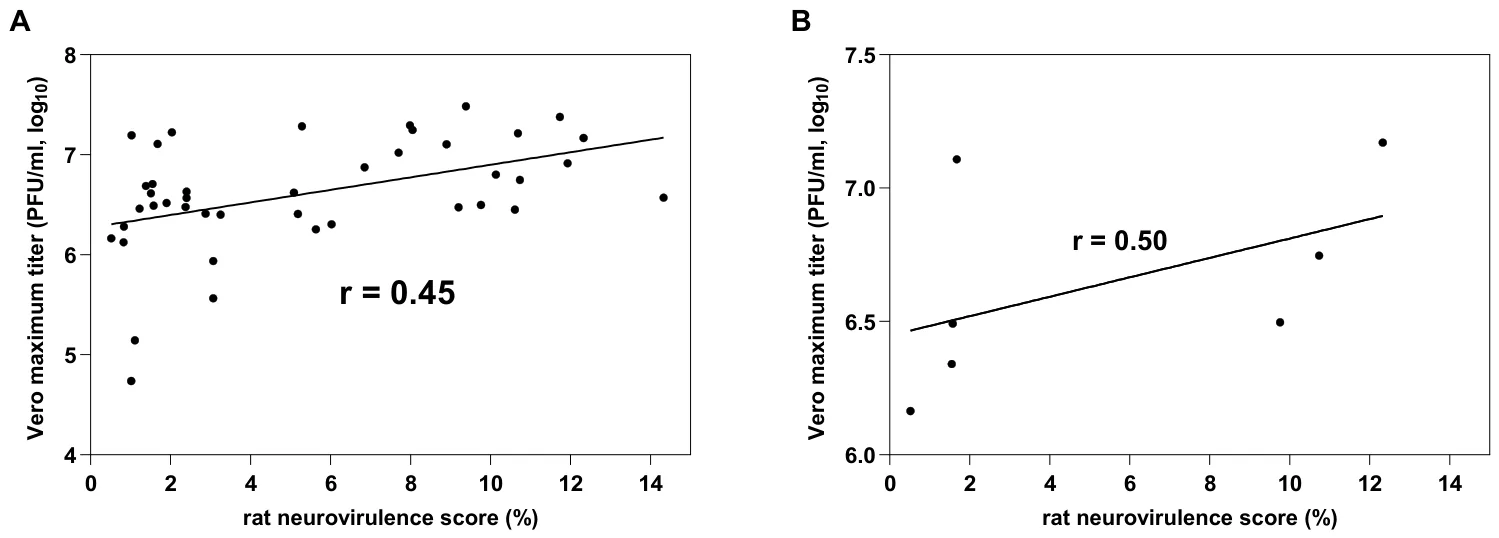
Correlation between rat neurovirulence scores and Vero maximum titers. (**A**): Neurovirulence scores (in %) of all 40 viruses for which growth kinetics in Vero cells were performed are plotted against peak virus titers in Vero cells (in pfu/mL, log_10_). In addition, neurovirulence scores and peak titers of non-recombinant viruses Urabe P-AM9 and Urabe P6-CEF [51] were included in the regression analysis. See Table 1 for information on peak virus titers for each virus and Figure S1 for depiction of growth kinetics for each virus. (**B**): Peak growth titers in Vero cells of only the seven viruses that were assessed for growth in brains (Figure 5) were plotted against their respective neurovirulence scores, and regression analysis was performed. Coefficients of correlation (r values) are indicated.

We also explored the correlation between virus neurovirulence in rats and virus growth in vitro to determine if the latter parameter could be used as an alternative to animals for assessing MuV neurovirulence. In Vero cells, based on a dataset of all 40 viruses for which growth kinetics were performed, the correlation between rat neurovirulence scores and peak virus titers in Vero cells was only moderate (Figure 6A, r = 0.45). A similar correlation was observed for the seven viruses whose growth kinetics were also analyzed in rat brains (Figure 6B, r = 0.5). These data indicate that Vero cells cannot be used to predict virus neurovirulence.

**Figure 7:**
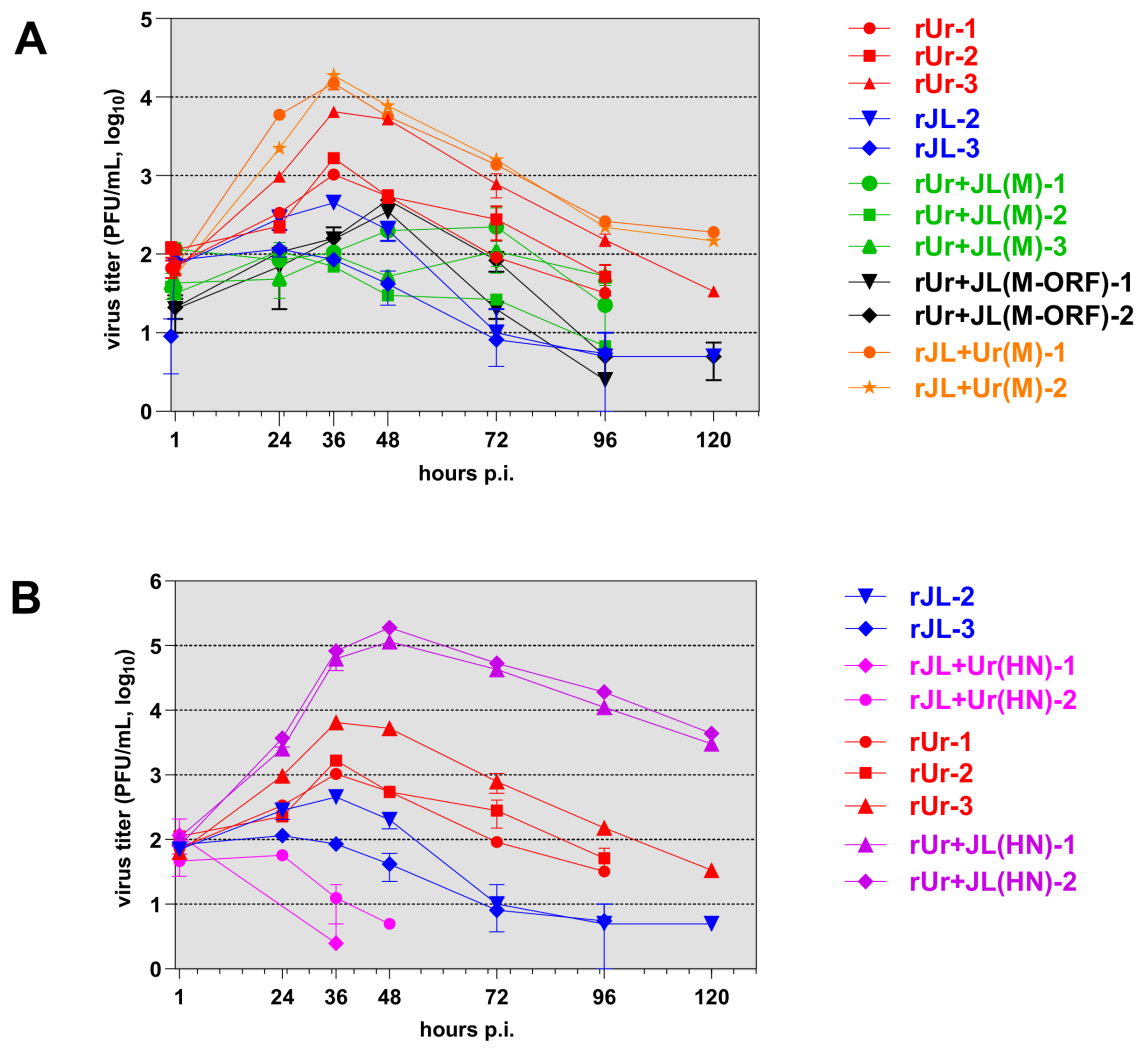
Multicycle growth kinetics of recombinant viruses in rat neuronal AF5 cells. Confluent monolayers of rat AF5 cells were inoculated at an MOI of 0.05, and supernatant samples were collected at the indicated time points post infection. (**A**): Growth kinetics of chimeric JL and Urabe AM9 viruses in which the M gene was swapped are compared to growth kinetics of non-chimeric JL and Urabe AM9 viruses. Virus titers per time point are provided in Table S5. (**B**): Growth kinetics of chimeric JL and Urabe AM9 viruses in which the HN gene was swapped are compared to growth kinetics of non-chimeric JL and Urabe AM9 viruses. Virus titers per time point are provided in Table S6. Note that growth kinetics that were conducted on two different occasions are depicted in the same panels (see the Material and Methods and Results sections for further information).

The correlation between rat neurovirulence (RN) scores and peak virus titers in vitro was also explored using the rat neuronal cell line AF5 [54]. Multi-cycle growth kinetics of the three rescued Urabe AM9 viruses and of two JL viruses were compared with those of the three rUr + JL(M) viruses, the two rUr + JL(M-ORF) viruses, and the two rJL + Ur(M) viruses. Growth of all viruses was much more restricted in AF5 cells compared to Vero cells (Figure 7A). In the first experiment, viruses rJL-3, rUr-1, rUr-2, rUr + JL(M)-2 and rUr + JL(M)-3 were evaluated. rJL-3 replicated very poorly, reaching a maximum titer of only 116 pfu/mL at 24 h post infection, whereas rUr-1 and rUr-2 replicated about one log_10_ PFU/mL higher (1047 pfu/mL and 1660 pfu/mL, respectively). Viruses rUr + JL(M)-2 and rUr + JL(M)-3 grew as poorly as rJL-3, reaching peak titers of only 110–107 pfu/mL (Figure 7A). These results were confirmed in a second experiment assaying viruses rJL-2, rUr-3, rUr + JL(M)-3, as well as viruses rUr + JL(M-ORF)-1 and rUr + JL(M-ORF)-2 (Figure 7A). While both rJL-2 and rUr-3 grew to slightly higher titers in this experiment compared to the other rJL and rUr viruses tested before (457 pfu/mL and 6456 pfu/mL, respectively) (Figure 7A), the difference between rJL-2 and rUr-3 viruses was still more than one log_10_ pfu/mL. Furthermore, rUr + JL(M)-3 as well as viruses rUr + JL(M-ORF)-1 and -2 grew to titers not higher than those observed for rJL-2 (389 pfu/mL, 347 pfu/mL and 490 pfu/mL, respectively). Finally, in the same experiment, viruses rJL + Ur(M)-1 and rJL + Ur(M)-2 were analyzed for growth in AF5 cells. As shown in Figure 7A, both viruses grew to higher titers compared to rUr-3 (1.5 × 10^4^ and 1.9 × 10^4^ PFU/mL, compared to 6.46 × 10^3^ pfu/mL). Given the absence of overt cytopathic effect (CPE) in infected cells, these data suggest that the Urabe M gene strongly promotes release of infectious virus from rat neuronal AF5 cells when placed in the JL backbone, whereas the JL M gene has the opposite effect when placed in the backbone of the Urabe AM9 virus.

In contrast to the Urabe AM9 M gene, the Urabe AM9 HN gene had a negative effect on the growth of JL- and Urabe AM9-based viruses in AF5 cells, as shown in Figure 7B. Both recombinant JL-based viruses in which the JL HN gene was replaced with the HN gene of Urabe AM9 (rJL + Ur(HN)-1 and 2) were not able to produce any infectious virus. On the other hand, recombinant Urabe AM9 viruses rUr + JL(HN)-1 and rUr + JL(HN)-2, in which the Urabe AM9 HN gene was replaced with that of JL, replicated to significantly higher titers compared to the three recombinant Urabe viruses, suggesting that the JL HN gene promotes a more efficient release of virus from rat neuronal cells, with the Urabe AM9 HN gene having the opposite effect. Overall, the correlation between RN scores and peak virus titers in AF5 cells for the viruses analyzed in Figure 7 was similar compared to that in Vero cells (r = 0.47), suggesting that established neuronal cell lines might not be useful as predictors for neurovirulence.

## 4. Discussion

The resurgence of mumps, despite high vaccine coverage, is attributed in part to genetic divergence between the vaccine strain and circulating wild-type strains [3,6,7,8,9,10]. This has led to calls for development of new live, attenuated, or subunit mumps vaccines [9,11,14,15,16]. However, a major issue with mumps vaccines has been their safety, as evidenced by the licensure of several MuV vaccines that have later been discovered to cause aseptic meningitis, leading to their withdrawal from the market [33]. The one clear exception has been the JL mumps vaccine strain, which is the most widely used vaccine strain globally, and no increased risk of aseptic meningitis has been linked to its use [31,33,37,62]. Approaches that have been considered for developing safe new live, attenuated mumps vaccines include replacement of the JL strain’s F and HN proteins, which are the targets of protective neutralizing antibodies [8], with those of the strain with the desired genotype [14,39] or genetic modification of wildtype isolates representative of currently circulating strains [9,15,44,45]. In the case of replacing JL genes with genes from wildtype viruses, assurance is required that the candidate JL-based vaccine virus has not gained neurovirulence, and in the case of modifying a wild-type virus, assurance is needed that the virus’ intrinsic neurovirulence properties have been eliminated. Prior studies involving replacement of the HN and F genes of JL with those from wild-type or lab-adapted strains, without additional genomic modifications, have reported different degrees of increased neurovirulence in a rat-based model relative to the highly attenuated parental JL strain, from modest increases to significant increases [14,39,41]. In the present study using the Urabe AM9 vaccine strain, individual replacement of the F gene, but not the HN gene, of JL with that of Urabe AM9 led to a slight but statistically significant increase in neurovirulence, which was increased further with the combined replacement of both the F and HN genes. Although individual replacement of the JL HN gene with the Urabe AM9 HN gene did not increase the neurovirulence of the JL-based chimeric virus, this virus rapidly formed large syncytia in Vero cells, with complete lysis by day 3 p.i. This resulted in the lowest viral growth observed among all JL–Urabe AM9 chimeric viruses analyzed in Vero cells and suggests a functional incompatibility between the JL F and Urabe HN proteins. A similar observation was made with the HN gene of strain 88-1961 in the JL backbone [39]. Therefore, while replacing only the HN gene of JL with the HN gene of a currently circulating MuV strain might yield viruses with similar neuroattenuation to that of JL, the viruses might exhibit poor replication, which is disadvantageous from a vaccine manufacturing perspective.

The second approach (genetic modification of a wild-type strain to achieve attenuation) has been met with better success. A significant neuroattenuating effect was reported with functional inactivation of the viral V gene, an important antagonist of the viral innate response [18,44]. Inactivation of the V gene without compromising the P gene was achieved by changing the editing site of the P/V/I gene from GGGGGG to GAGGAG and adding the two Gs that are required for proper translation of the P protein. The V-deficient wild-type virus was shown to be highly attenuated, comparable to the level observed with the JL vaccine [44]. However, in a prior study, replacing the V gene of a wild-type strain (88-1961) with the V gene of Jeryl Lynn had no attenuating effect on the resulting chimeric virus [39]. This was also the case in the present study: Combined replacement of the N, V/P/I and L genes of Urabe AM9 with those of JL (in the form of virus JL + Ur(M/F/SH/HN)) did not have a neuroattenuating effect. This suggests that the highly attenuated phenotype of the JL vaccine strain is not due to genetic differences between the JL V gene and the V genes of the 88-1961 and Urabe AM9 strains. Thus, while the V protein is required for a strain’s virulence, it is not sufficient to confer virulence to a strain that has acquired attenuation through mutations elsewhere in the genome. That functional inactivation of V can happen naturally or empirically in vitro is unlikely, given that multiple mutations and an insertion were required to achieve V gene inactivation. Nevertheless, it should be noted that in the present study, we replaced genes instead of inactivating them, so we cannot comment on the possibility that the observed effect of V gene inactivation reported by others is broadly applicable.

Deletion of the SH gene has also been shown to lead to partial attenuation of recombinant wildtype viruses such as the 88-1961 strain [28] or the MuV-Iowa (IA) strain [26] and has been combined with the inactivation of the V gene in another study to accomplish full neuroattenuation [14]. Notably, while such deletion has thus far not been reported to occur naturally, point mutations in the F polyadenylation signal have been observed for several MuV strains, including the Enders strain, leading to the absence of monocistronic SH mRNAs and subsequent lack of SH synthesis [27]. However, the finding that neurovirulence is unaffected by the functional inactivation of the SH gene via stop codons suggests that the SH protein itself is not a determinant of neurovirulence [28]. This is further supported by the studies in which the JL SH gene was replaced by the SH gene of the Kilham strain or the 88-1961 strain without any significant effect on neurovirulence [39,41]. While we did not individually replace the JL SH gene with that of Urabe AM9, individual replacement of the Urabe AM9 SH gene with that of JL did not significantly change the neurovirulence of the resulting chimeric virus. In the present study, replacement of three or more genes was required to either confer neurovirulence to JL or fully attenuate the Urabe AM9 virus. Accordingly, neither combined replacement of the JL F and HN genes with those of Urabe AM9 nor replacement of the Urabe AM9 F and HN genes with those of JL fully conferred or abrogated neurovirulence in the resulting viruses, respectively, similar to our findings from studies with the 88-1961 strain [39]. In contrast, replacing the Urabe F and HN genes together with the SH gene led to a significant further decrease in neurovirulence, and replacing the Urabe AM9 F and HN genes together with the Urabe AM9 M gene (with or without the Urabe AM9 SH gene) with those of JL led to the greatest reduction in neurovirulence. Similarly, the combined replacement of the JL M, F, SH and HN genes with those of Urabe AM9 in all three rJL + Ur(M/F/SH/HN) viruses was sufficient to confer a neurovirulent phenotype on the JL virus, indicating that the Urabe AM9 proteins associated with viral replication, i.e., N, P, and L, are not determinants of neurovirulence for Urabe AM9. Consistent with this observation, replacement of the JL L gene with that of Urabe AM9 did not lead to a biologically meaningful increase in neurovirulence of virus rJL + Ur(L). To this end, we cannot exclude the possibility that other combinations of two or more JL genes not evaluated in this study could confer complete attenuation of Urabe AM9. However, these data, together with our previous study with 88-1961, indicate that both the neurovirulence of MuV wildtype strains and the neuroattenuation of the JL vaccine are multigenic traits. Of note, using a similar approach to generate chimeric Newcastle disease viruses (NDV, a close relative of MuV), only the simultaneous replacement of the M, F and HN genes from one pathogenic NDV strain could impart its phenotype on another NDV strain with different pathogenic features and vice versa [63]. It should be noted that while the Urabe M, F, SH and HN genes in combination were sufficient to confer neurovirulence to the same extent as that of the Urabe AM9 virus, this was not the case for the 88-1961 wildtype virus. The fact that the N, P and L genes of JL and Urabe AM9 are interchangeable without a significant biological effect on attenuation suggests that these genes accumulated attenuating mutations during repeated passages in the different avian and/or monkey substrates. In contrast, the N, P and L genes of the wildtype 88-1961 strain may have maintained neurovirulent potential, presumably since the virus was not exposed to extensive serial passaging in a different cell substrate and thus no substrate-specific adaptive mutations could occur. Whether the N, P and L genes of other MuV wildtype isolates similarly behave like those of MuV strain 88-1961 remains to be investigated. Finally, it should be emphasized that our conclusion that MuV neurovirulence is a multigenic trait is based on swapping studies with the empirically attenuated Jeryl Lynn strain only. Thus, we cannot formally exclude the possibility that empirical attenuation of other MuV isolates by means of serial passaging could be due to a single mutation, even though this has not been demonstrated thus far, as discussed above. The most significant effect of a single gene replacement in the Urabe AM9 genome was observed with the JL M gene, with a decrease in the average neurovirulence score by 70% compared to that of the Urabe AM9 virus. The neuroattenuating effect of the JL M gene was also found in our previously reported study of the wildtype 88-1961 virus (although the decrease in neurovirulence was not as pronounced) [39]. Conversely, replacement of the JL M gene with that of neurovirulent MuV strains Urabe AM9, 88-1961, or Kilham increased the rat NV score of the chimeric viruses by factors of 2.1, 2.3, and 2.8, respectively, as shown in this study and previously [39,41], indicating an important role of the MuV M gene both in neurovirulence and neuroattenuation. This M gene effect on attenuation was also reported with canine distemper virus, a member of the *morbillivirus* genus within the *paramyxoviridae* family [64], demonstrating the importance of the M gene for virulence. The attenuating effect of the JL M gene could be due to differences in interactions between the M protein and its viral counterparts or cellular proteins, or both.

Paramyxovirus M proteins function as central orchestrators in virus assembly and budding by serving as adapters that link ribonucleoproteins and viral glycoproteins at specific plasma membrane sites [22,65]. For MuV, efficient virus-like particle (VLP) production requires M, N and F proteins [23], with the M protein containing a conserved FPVI (aa 24–27) motif that acts as a late domain essential for recruiting the host ESCRT machinery to budding sites. The late domain of the M protein of NDV interacts with CHMP4B, part of the ESCRT subcomplex III [66], and dominant mutants of both CHMP4B and Vsp4 inhibit MuV VLP formation [23], suggesting that CHMP4B also interacts with the FPVI motif of MuV. Of note, the M proteins of the JL and Urabe AM9 strains differ at only five aa positions (M28V, Q53H, Y140H, I207V, R373S). Since the M28V change is located adjacent to the FPVI motif, it will be interesting to analyze whether this change affects CHMP4B binding and virus budding.

The MuV M protein employs an additional ESCRT-III recruitment strategy by binding to angiomotin-like 1 (AmotL1), which bridges the M protein to E3 ubiquitin ligases through PPXY sequences, facilitating ubiquitination and subsequent ESCRT-III recruitment [67]. The M protein shuttles between the nucleus and cytoplasm via bipartite nuclear localization and nuclear export signals [68], with nuclear accumulation affecting host protein synthesis. Ubiquitination at specific lysine residues is crucial for nuclear export and proper budding. Though the aa changes between the JL and Urabe AM9 M proteins do not directly affect identified ubiquitination sites, indirect effects such as steric hindrance cannot be excluded. Furthermore, the aa of MuV M that mediate binding to AmotL1 have not yet been delineated, and binding might be affected by aa changes between the JL and Urabe AM9 M proteins. Recent studies identified Treacle as another nuclear interaction partner of the MuV M protein, important for efficient late-stage viral growth [69], though the critical aa for this interaction remain unmapped.

Taken together, it is tempting to speculate that qualitative and/or quantitative differences in protein–protein interactions between the M proteins of neuroattenuated versus neurovirulent strains and cellular proteins might exist that could possibly explain the observed effects on neurovirulence in the different chimeric viruses. However, these effects might also be due to changes in the interactions between M and other viral proteins. While paramyxovirus glycoproteins bind M proteins through their cytoplasmic tails and the MuV N protein interacts via its conserved C-terminal DWD motif with MuV M [70], the specific MuV M protein domains responsible for these interactions have not been fully characterized. Future studies are therefore warranted to map the aa in the M protein that are critical for interaction with its cellular partners and with viral N and HN proteins. Notably, the M proteins of JL and strain 88-1961 differ in five aa positions, with three of these changes being identical between Urabe AM9 and 88-1961 (Y140H, I207V, and R373S). Therefore, future studies should investigate whether these modifications alone or in combination have an effect on cellular and viral partner interactions and whether any of these changes may be determinants of neurovirulence.

Previous studies using chimeric viruses between the JL and 88-1961 MuV strains demonstrated a strong correlation between neurovirulence scores and viral growth efficiency in rat brains [39]. This study confirmed this correlation using multiple chimeric viruses and showed that replacing the Urabe M gene with JL’s decreased chimeric virus growth compared to that of parental Urabe viruses. Evidence suggests that attenuated MuV strains like JL only infect the ependymal cell lining, the predominant site of viral replication following intracerebral inoculation of rats, whereas neurovirulent strains such as 88-1961 can spread from ependymal cells to surrounding brain parenchymal neurons [71]. This suggests that neurovirulent strains exhibit a stronger tropism for neurons. While this hypothesis requires further study, using the neuronal cell line AF5, recombinant JL viruses showed severely impaired infectious progeny release following infection, while recombinant Urabe AM9 viruses grew more efficiently. Critically, all chimeric Urabe viruses with M gene or M ORF replacement by JL showed reduced AF5 growth to JL virus levels. Conversely, replacing the JL M gene with the Urabe AM9 gene in rJL + Ur(M) viruses significantly increased AF5 titers, emphasizing the importance of the Urabe M gene for efficient neuronal cell growth. These chimeric viruses achieved titers exceeding even recombinant Urabe viruses, clearly demonstrating the M gene’s crucial role in neurovirulence.

In a study by Ninomiya et al. [72], the M gene of the attenuated Hoshino strain was replaced with that of the clinical Ur89-250 isolate (Urabe AM9 strain). The chimeric virus replicated efficiently in SH-SY5Y neuroblastoma cells, which support growth of the Urabe strain (specifically Ur89-250) but not the Hoshino vaccine strain, demonstrating that the Ur89-250 M gene confers the ability to replicate in neuronal cells. These findings further implicate the M protein as a determinant of MuV neurovirulence, possibly by affecting neurotropism, and underscore the need to define the specific amino acids responsible for this phenotype.

A major limitation of this and similar studies assessing the contribution of individual MuV genes to neurovirulence using recombinant viruses is the frequent occurrence of unintended second-site mutations arising during virus rescue in BHK-BSR-T7 cells and amplification in Vero cells, as shown in this study and previously [38,39,59]. These variants typically appear as single-nucleotide heterogeneities (SNH) in coding or noncoding regions or as single-nucleotide insertions in homopolymeric tracts at gene-end signals. Of the 41 recombinant viruses examined, only seven lacked variants exceeding 10% of the viral population; the remainder carried one to five such SNHs, and ten viruses contained SNHs or insertions present in more than 50% of the genomes.

Although the effect of these individual mutations on neurovirulence is unknown, earlier work showed that changes in the genetic heterogeneity of the Urabe AM9 strain can markedly alter neurovirulence [51]. Consistent with this, plaque-purified variants of the L-Zagreb and Urabe AM9 strains differed in mutational profiles and displayed distinct levels of neuroattenuation in animal models [40,42]. Studies of wild-type strain 88-1961 likewise suggested that single-nucleotide mutations in N, M, L, F, and HN can modulate neurovirulence [43,60,71], though incomplete sequencing in those experiments leaves open the possibility that additional second-site mutations may also have contributed. These observations underscore the difficulty of distinguishing the effects of intentional genetic modifications (point mutations or gene swaps) from those of unintended mutations elsewhere in the genome. Swapping genes between MuV genotypes may impair efficient viral replication, assembly and budding and might force selection for compensatory mutations that mitigate the effects on growth. Specifically, 11 SNHs were identified in the L gene that exceeded the threshold of 50%. Whereas one was a silent substitution, ten were predicted to result in amino acid changes, four of which were conservative and six of which were radical changes (Table S1). Three of the four conservative aa changes are localized in blocks identified by Sidhu et al. [73] and Poch et al. [74] as being highly conserved among L protein polymerases of non-segmented negative-strand (NNS) RNA viruses. Two of these changes (L1167V in rJL + Ur(M/F/SH/HN)-2 and L1307V in rJL + Ur(M/F/SH/HN)-3) are localized within block V, with a hydrophobic aa being the preferred aa occupying aa 1307. The difference in NV scores of viruses rJL + Ur(M/F/SH/HN)-2 and -3, which is statistically significant (14.33 vs. 6.85; Table 1), could be related to the functional consequences of these sequence changes in the L protein between these two variants. The other conservative aa change (I217V) in virus rUr + JL(M/F/SH/HN)-1 is localized within block I, but isoleucine 1217 is not conserved among L proteins of different NNS RNA viruses. Three of the six radical aa changes in the L protein also were localized within the blocks of high similarity with other L proteins of NNS RNA viruses. The radical aa change P544H in virus rUr + JL(F)-3 is located within block II, with proline 544 being highly conserved. Finally, adjacent nucleotide changes C9636A and C9637G in virus rUr + JL(HN)-3 both had almost similar prevalence, suggesting that they were localized on the same RNA strand. Irrespective of whether the two mutations are linked, there is a predicted radical change in aa T400 in block I of the L protein to either N400, L400, or K400 (Table S1). However, T400 is not highly conserved among L proteins of different non-segmented negative-strand (NNS) RNA viruses.

Of note, Vero growth curves of independently rescued viruses from the same cDNA were not always identical, likely reflecting these second-site mutations.

To minimize confounding by second-site mutations, at least two independently rescued viruses were evaluated for each cDNA, as was done in previous work [39]. No recurring mutations were found across independent rescues, supporting their stochastic origin. We therefore reasoned that it is unlikely that different mutations in two rescued viruses would have similar effects on neurovirulence phenotypes. Most independently rescued viruses yielded similar neurovirulence scores; when scores differed substantially, a third virus was tested. In several cases, up to six independent rescues were sequenced, and viruses containing near-fixed nonsynonymous SNHs were excluded. Collectively, these results emphasize the necessity of sequencing every recombinant MuV and, when unwanted mutations are present, testing multiple independently rescued viruses. Reliance on a single rescued stock risks misinterpretation. Finally, although low-frequency SNHs might gain a selective advantage during replication in the newborn rat brain, logistical and ethical constraints precluded sequencing of rat-brain-derived viruses for this study.

The rat NV assay, while considered the gold standard for testing MuV vaccine neurotoxicity, has significant limitations due to the length of time until completion and, in particular, the high variability in neurovirulence scores between and within litters. This variability is unexplained and unrelated to sex differences, dose, or inoculation timing [75]. It is therefore challenging to detect more subtle differences in neurovirulence. To address this variability, it is essential to test at least three litters per virus, with each litter consisting of seven or more pups. However, the requirement to test multiple virus rescues to mitigate the effects of second-site mutations necessitates large numbers of animals, raising ethical concerns and highlighting the need for in vitro alternatives. Moreover, since viruses are directly inoculated into the brain, the assay only allows assessment of neurovirulence, but not neuroinvasiveness, of a given virus, which is another limitation of the assay.

Our previous study [39] and the current study demonstrated a correlation between neurovirulence scores and maximum viral titers in infected newborn rat brains, suggesting that in vitro virus growth in a suitable substrate could serve as a surrogate marker for neurovirulence. However, testing revealed only a moderate correlation between peak virus titers in Vero cells and neurovirulence scores (*r* = 0.45 in the previous study [39] and *r* = 0.5 in this study), indicating that Vero cells are unsuitable as replacement models. The reason for the poor correlation between growth in Vero cells and rat brains is unknown but points to mechanisms of virus attachment, cell entry and replication that are specific to neuronal cells. Evaluation of the rat neuronal cell line AF5 showed promise, as chimeric viruses rUr + JL(M) and rUr + JL(M-ORF) replicated poorly in AF5 cells, consistent with their reduced brain replication and neurovirulence. However, with respect to virus rJL + Ur(M), the correlation was imperfect. Additionally, replacing the Urabe HN gene with JL’s resulted in enhanced AF5 growth compared to the parental rUr viruses, but no corresponding increase in neurovirulence, suggesting that AF5 cells may not ideally recapitulate newborn rat brain viral kinetics. The specific amino acid responsible for increased growth of JL HN in AF5 cells remains unknown, though previous research identified the N464S change as critical for efficient growth in SH-SY5Y cells [72]. Intriguingly, there is a N464H change in the HN protein of JL at this position compared to the Urabe AM9 HN protein, and it remains to be investigated whether this change in the chimeric virus is sufficient to confer increased neurotropism in AF5 cells. Since AF5 cells are immortalized cells, the presented results need to be interpreted with caution, and it remains to be shown whether the observations made are specific to this particular cell line or can be recapitulated using primary rat neurons. Moreover, a detailed analysis of the cellular tropism of neurovirulent vs. neuroattenuated virus strains in the newborn rat brain is needed and will be instrumental in informing the choice of cell substrates suitable to replace the rat neurovirulence test. Besides established cell lines, primary cell cultures and/or rat brain organotypic slice cultures should be considered to reduce animal use while maintaining predictive accuracy.

Together with previous studies, a common theme appears to emerge, indicating that full MuV neurovirulence or complete neuroattenuation is due to a combination of markers in different genes rather than to one single marker, with the M gene playing a central role. Given this role in neurovirulence, with respect to future vaccine development, manipulation of both the M and V genes should be considered to be key for added assurance of attenuation. The findings presented here are of relevance for the rational design of novel live attenuated MuV vaccines and highlight the need to develop reliable in vitro models to test for neurovirulence.

## 5. Conclusions

In this study, we demonstrated that the MuV M gene plays a dominant role in determining the neurovirulence phenotype. Replacement of the Urabe AM9 M gene with that of the highly attenuated JL strain resulted in a statistically and clinically significant 70% reduction in neurovirulence, the most substantial single-gene effect observed. Combined replacement of the Urabe M, F, SH, and HN genes with their JL counterparts nearly completely attenuated the neurovirulent Urabe AM9 virus, while the reciprocal swap conferred full neurovirulence to JL, indicating that these four genes are necessary and sufficient to determine the neurovirulent phenotype.

Our findings reveal that neurovirulence is a multigenic trait in which the M gene exerts a dominant effect, while the F and HN genes contribute additively. Notably, the JL M gene had a more pronounced attenuating effect on the Urabe background than the Urabe M gene had on the JL background, suggesting that neurovirulence and neuroattenuation involve distinct genetic mechanisms. The M protein’s central role likely reflects its essential functions in viral assembly and budding; the five amino acid differences between JL and Urabe M proteins, particularly the M28V change adjacent to the conserved FPVI late domain motif, may affect interactions with host ESCRT machinery and other cellular factors critical for neurotropism. In addition, effective binding of JL M to its Urabe AM9-derived binding partners, such as the nucleoprotein and glycoproteins, might be affected, which warrants further investigation.

Our study also provides important methodological insights for future vaccine development studies. The identification of single-nucleotide heterogeneities (SNHs) in the majority of independently rescued recombinant viruses underscores the critical importance of sequencing each viral clone, testing multiple independent rescues, and implementing multi-tiered validation approaches. The high frequency of second-site mutations that arose during rescue and propagation highlights inherent limitations of recombinant virus systems and the necessity for rigorous quality control in comparative studies of viral genetics. Such rigorous quality control is also of utmost importance to avoid the potential risk of virulence reversion associated with these SNHs.

For the rational design of next-generation mumps vaccines, these findings suggest that any strategy to attenuate a wild-type strain should include replacement of the M gene with that of JL, in addition to targeting other genes, such as the V gene. The identification of the M gene as a critical modulator of neurovirulence provides evidence-based guidance for developing safer live, attenuated mumps vaccines with improved safety profiles, which is particularly important given the current resurgence of mumps in vaccinated populations and the need for vaccines with improved immunogenicity and durability.

## Figures and Tables

**Table 1:** Comparison of rat neurovirulence scores and peak virus titers in Vero cells of individual rescued viruses.

Virus	Rescue	N	RNV Score ^i^ (Mean ± SD)	*p*-Value ^j^	Max Titer Vero (pfu/mL, log_10_) and Day Post-Infection When Max Titer Was Reached ^a^
rJL	1	37	0.86 ± 1.02 ^b^	*p* = 0.28 (res 2 vs. res 1)	6.28 d2 ^d^
	2	7	0.52 ± 0.24 ^b,c^	*p* = 0.681 (res 3 vs. res 2)	6.16 d2 ^e^
	3	9	0.78 ± 0.73 ^c^	*p* = 0.621 (res 3 vs. res 1)	nd
rJL + Ur(M)	1	29	1.88 ± 2.17	*p* = 0.5105	6.52 d2
	2	28	1.51 ± 2.03		6.62 d2
rJL + Ur(F)	1 ^f^	24	1.39 ± 1.24	***p* = 0.017 (res 2 vs. res 1)**	6.69 d4
	2	27	**1.03 ± 1.65**	***p* = 0.02 (res 3 vs. res 2)**	7.19 d4
	3 ^f^	27	**2.4 ± 2.47**	*p* = 0.298 (res 3 vs. res 1)	6.57 d4 ^g^
rJL + Ur(HN)	1	23	0.55 ± 0.31	*p* = 0.14	5.15 d2
	2	20	1.25 ± 1.95		4.74 d2
rJL + Ur(L)	1	34	0.83 ± 0.52	N/A	6.12 d2
rJL + Ur(F/HN_T577A_)	1	25	3.07 ± 3.3	*p* = 0.669	5.56 d4 ^h^
	2	37	3.35 ± 3.4		5.94 d4
rJL + Ur(M/F/SH/HN)	1	24	**10.14 ± 5**	***p* = 0.0198 (res 2 vs. res 1)**	6.8 d3
	2	24	**14.33 ± 6.87**	***p* = 0.0009 (res 3 vs. res 2)**	6.57 d3
	3	25	6.85 ± 6.37	*p* = 0.0735 (res 3 vs. res 1)	6.87 d3
rUr	1	25	12.34 ± 6.76	*p* = 0.206 (res 2 vs. res 1)	7.17 d3
	2	32	9.76 ± 8.07	*p* = 0.668 (res 3 vs. res 2)	6.5 d3
	3	21	**10.69 ± 6.94**	*p* = 0.421 (res 3 vs. res 1)	7.21 d2
rUr + JL(M)	1	26	1.58 ± 1.76	***p* < 0.0001 (res 2 vs. res 1)**	6.49 d3
	2	23	6.03 ± 4.29	***p* = 0.005 (res 3 vs. res 2)**	6.3 d3
	3	31	2.88 ± 2.26	***p* = 0.007 (res 3 vs. res 1)**	6.41 d3
rUr + JL(M-ORF)	1	33	5.19 ± 5.57	*p* = 0.339	6.41 d3
	2	24	3.25 ± 3.39		6.4 d3
rUr + JL(F)	1	19	5.64 ± 5.23 ^b^	***p* = 0.009 (res 2 vs. res 1)**	6.26 d3
	2	24	**10.61 ± 6.04**	*p* = 0.45 (res 3 vs. res 2)	6.45 d2
	3	19	**9.19 ± 6.05**	*p* = 0.08 (res 3 vs. res 1)	6.47 d3
rUr + JL(SH)	1	16	**7.71 ± 6.54**	*p* = 0.583	7.02 d3
	2	20	**8.89 ± 6.29**		7.11 d2
rUr + JL(HN)	1	22	9.39 ± 6.32	*p* = 0.287 (res 2 vs. res 1)	7.48 d3
	2	24	8.05 ± 4.69	*p* = 0.061 (res 3 vs. res 2)	7.25 d3
	3	20	**11.74 ± 5.87**	*p* = 0.372 (res 3 vs. res 1)	7.38 d4
rUr + JL(F/HN)	1	23	**7.99 ± 5.69** ^b^	***p* = 0.049 (res2 vs. res1)**	7.29 d3 ^e^
	2	28	5.09 ± 5.20	***p* = 0.0002 (res 3 vs. res 2)**	6.62 d2
	3	29	**11.93 ± 7.44**	***p* = 0.0413 (res 3 vs. res 1)**	6.91 d3
rUr + JL(F/SH/HN)	1	55	2.01 ± 3.67	*p* = 0.253	7.22 d2
	2	26	5.29 ± 6.81		7.28 d2
rUr + JL(M/F/HN)	1	30	1.23 ± 0.81	*p* = 0.074	6.46 d2
	2	24	2.38 ± 2.78		6.48 d2
rUr + JL(M/F/SH/HN)	1	24	1.68 ± 1.61	*p* = 0.859	7.11 d3
	2	27	2.4 ± 4		6.63 d2

^a^: Unless stated otherwise, all kinetics experiments were done in duplicate in a 6-well plate format. ^b^: Historical data were used: rJL-1 and rJL-2 [39]; rUr + JL(F)-1 and rUr + JL(F/HN)-1 [58]. ^c^: Only one rat litter was inoculated and analyzed. ^d^: Mean from three independent kinetic experiments, each carried out in one T75 cm^2^ flask, and from one kinetics experiment carried out in duplicate in a 6-well plate format. ^e^: Mean from two kinetics experiments carried out at different times, each in 6-well plates. ^f^: To generate rJL + Ur(F)-1 and 3, aliquots from the same supernatant harvested from one rescue assay in BHK-BSR T7 cells were used to infect two different Vero cell passages. For virus stock 3, infected Vero cells and virus-containing supernatant were sonicated, followed by centrifugation. For virus stock 1, the supernatant of infected Vero cells was harvested without subsequent sonication. ^g^: Mean from two independent kinetics experiments, each using one T75 cm^2^ flask. ^h^: Kinetics experiment carried out in one T75 cm^2^ flask. ^i^: Rat NV data for each individual rescued virus were subjected to statistical normality testing using the Shapiro–Wilk test. Datasets that passed the normality test (a = 0.05) are highlighted in bold letters. ^j^: For comparison of datasets that passed the normality test, an unpaired *t*-test was employed. For datasets that did not pass the normality test, data were compared using the Mann–Whitney test. *p* values < 0.05 denote a significant difference (highlighted in bold letters). nd: not determined; max: maximum; N/A: not applicable. N = total number of rats analyzed per rescued virus.

**Table 2:** Number of recombinant viruses with identified variants at different prevalence thresholds ^a^.

Number of Variants per Virus	Number of Viruses (Variant Prevalence ≥ 10%)	Number of Viruses (Variant Prevalence ≥ 50%)
1	7	7
2	8	2
3	9	1
4	6	0
5	2	0
6	2	0
>6	0	0
	Number of viruses without variants with prevalence ≥ 10%	Number of viruses without variants with prevalence ≥ 50%
	7	31
Total Viruses Analyzed	41	41

^a^: Variants include single-nucleotide substitutions and insertions. No deletions were identified.

## Data Availability

All Fastq files of the HiSeq and MiSeq sequencing data used for this manuscript are openly available at http://www.ncbi.nim.nih.gov/bioproject/1483926. Files with the results of single-nucleotide polymorphism analysis for each virus sequenced by HTS using the FDA HIVE platform tools HIVE-hexagon and HIVE-heptagon will be made available by the authors on request. Individual rat hydrocephalus scores that are graphically depicted in Figures S3 and S5 are provided in Supplemental Tables S2 and S3, respectively. Brain virus titers for individual rat brains depicted in Figure 5A are provided in Supplemental Table S4. Virus titers for each virus and time point from multicycle growth kinetics of recombinant viruses in rat neuronal AF5 cells (as depicted in Figure 7A,B) are provided in Supplemental Tables S5 and S6, respectively. Scans of the brain sections analyzed in this study as well as hydrocephalus calculations for each brain will be made available by the authors on request.

## Supplementary Materials

The following supporting information can be downloaded at: https://www.mdpi.com/article/10.3390/vaccines14090774/s1, Supplemental Document: Generation of recombinant chimeric mumps virus cDNA constructs; Supplemental Figure S1: Multicycle growth kinetics of recombinant MuV in Vero cells. Confluent monolayers of Vero cells were infected with the indicated viruses at an MOI of 0.05 and incubated for 1 h. Cells were washed twice with PBS and fresh growth medium was added. Samples were taken at the indicated time points post-infection. Removed volumes were replaced with the same volumes of fresh growth media. Note that for some viruses, growth kinetics conducted at different times were shown in the same panel (see Table 1 for information). Furthermore, note that viruses Urabe P-AM9 and Urabe P6-CEF are non-recombinant MuV preparations described previously [51]; Supplemental Figure S2: Rat neurovirulence score calculations. The neurovirulence (NV) score measures the severity of MuV-induced hydrocephalus and is determined by calculating the cross-sectional areas of the brain (excluding the cerebellum) and the lateral ventricle on each tissue section using Image Pro Plus or ImageJ image analysis software. Left panels show sections of the left brain hemispheres from rats infected with JL vaccine virus, Urabe AM9 vaccine virus, or wildtype MuV strain 88-1961. Red arrows indicate the lateral ventricles. Cross-sectional area values for the lateral ventricles and brains (excluding the cerebellum) are indicated for each section. The hydrocephalus score is calculated as the ratio of these two measurements, expressed as a percentage. Hydrocephalus scores < 1% indicate full attenuation, while scores up to 30% are typical for highly neurovirulent wildtype isolates. Urabe AM9 virus is insufficiently attenuated, exhibiting a neurovirulence score of approximately 12%. The right panel depicts hydrocephalus score measurements from both the left and right brain hemispheres of rat 4486 infected with rUr + JL(HN)-2. Red arrows indicate the lateral ventricles, and fine yellow lines delineate the areas of the lateral ventricles and brains (excluding the cerebellum) measured with image analysis software. Green numbers (47–50) denote the measured lateral ventricles or brains for each hemisphere; corresponding values for these numbers are indicated. Hydrocephalus scores were calculated for both hemispheres, and the final score for rat 4486 represents the mean of scores from both brain hemispheres; Supplemental Figure S3: Rat NV scores of recombinant chimeric viruses in which genes of recombinant MuV vaccine strain Urabe AM9 (rUr) were replaced individually or in combination with corresponding genes from recombinant MuV vaccine strain JL (rJL). Bars represent the mean (±SD) NV score (in percent) of all rats inoculated with virus stocks generated from individual rescue events. See Table 1 for additional information on mean rat NV scores of individual rescued viruses depicted in this graph. See Table S2 for individual rat NV scores of all rats depicted in Figure S3; Supplemental Figure S4: Detailed graphical depiction of the differences between plasmids pUr + JL(M) and pUr + JL(M-ORF). Urabe AM9-specific genomic regions are shown in green color; JL-specific genomic regions are shown in blue color. Numbers in parentheses indicate genomic positions. The untranslated regions (UTRs) flanking the P, M and F genes are shown in more detail. The 3′UTRs each contain gene-end signals, while the 5′UTRs each contain gene-start signals. Gene-end and gene-start signals are separated by intergenic regions (here indicated by solid black bars separating the gene-end and gene-start signals). Note that in pUr + JL(M), nucleotides 3171–3264 of the P/M UTR are JL-specific and encompass nucleotides 3177–3226 of the P gene 3′UTR as well as nucleotides 3228 to 3264 of the M gene 5′UTR. Likewise, nucleotides 4392–4461 of the M gene 3′UTR are JL-specific, while nucleotides 4465–4481 of the M 3′UTR are Urabe AM9-specific. Finally, the F gene 5′UTR is Urabe AM9-specific. In pUr + JL(M-ORF), the P gene 3′UTR, the M gene 5′UTR and 3′UTR, as well as the F gene 5′UTR are all Urabe AM9-specific. The percentage of nucleotide changes in the UTR between the JL and Urabe AM9 strains is indicated; Supplemental Figure S5: Rat NV scores of recombinant chimeric viruses in which genes of recombinant MuV vaccine strain JL (rJL) were replaced individually or in combination with corresponding genes from recombinant MuV vaccine strain Urabe AM9. Bars represent the mean (±SD) NV score (in percent) of all rats inoculated with virus stocks generated from individual rescue events. See Table 1 for additional information on mean rat NV scores of individual rescued viruses depicted in this graph. See Table S3 for individual rat NV scores of all rats depicted in Figure S5; Supplemental Figure S6: Kinetics of syncytium formation in Vero cells infected with rJL and rJL + Ur(HN) viruses. Vero cells (12-well plate format) were infected at an MOI of 0.05 for one hour with the indicated virus or with medium (without FBS) alone. Following removal of the inoculum and washing of the cells, one mL of growth medium was added per well. Pictures were taken on days 1, 2 and 3 post-infection (p.i.) using light-field microscopy (20× magnification; Leica Axiovert 40 CFL microscope (Carl Zeiss Microscopy GmbH, Jena, Germany) and a ZEISS Axiocam 305 mono camera. Pictures were processed using ZEISS ZEN software version 3.8.99.02000; Supplemental Table S1: Allelic variants ≥ 10% identified in rescued viruses; Supplemental Table S2: Individual rat neurovirulence scores for all viruses shown in Figure S3; Supplemental Table S3: Individual rat neurovirulence scores for all viruses shown in Figure S5; Supplemental Table S4: Mean brain viral loads per day post-infection for all viruses shown in Figure 5; Supplemental Table S5: Mean viral titers per time point and virus in AF5 cells for all viruses shown in Figure 7A; Supplemental Table S6: Mean viral titers per time point and virus in AF5 cells for all viruses shown in Figure 7B.

## Abbreviations

The following abbreviations are used in this manuscript:

MuV	Mumps virus
JL	Jeryl Lynn
M	Matrix
NV	Neurovirulence
SNH	Single-nucleotide heterogeneity
HTS	High-throughput sequencing
pfu	Plaque-forming unit
SH	Short hydrophobic
