# Supplementary material for: A Chimeric Virus Approach Reveals the Matrix (M) Gene as a Critical Modulator of Mumps Virus Neurovirulence
Source: Vaccines (Basel). 2026 Sep 3;14(9):774. doi: 10.3390/vaccines14090774 (PMC13611789; doi:10.3390/vaccines14090774)
Supplement: Supplementary file 1 [file vaccines-14-00774-s001.zip › Supplemental Figures S1-S6.pdf]

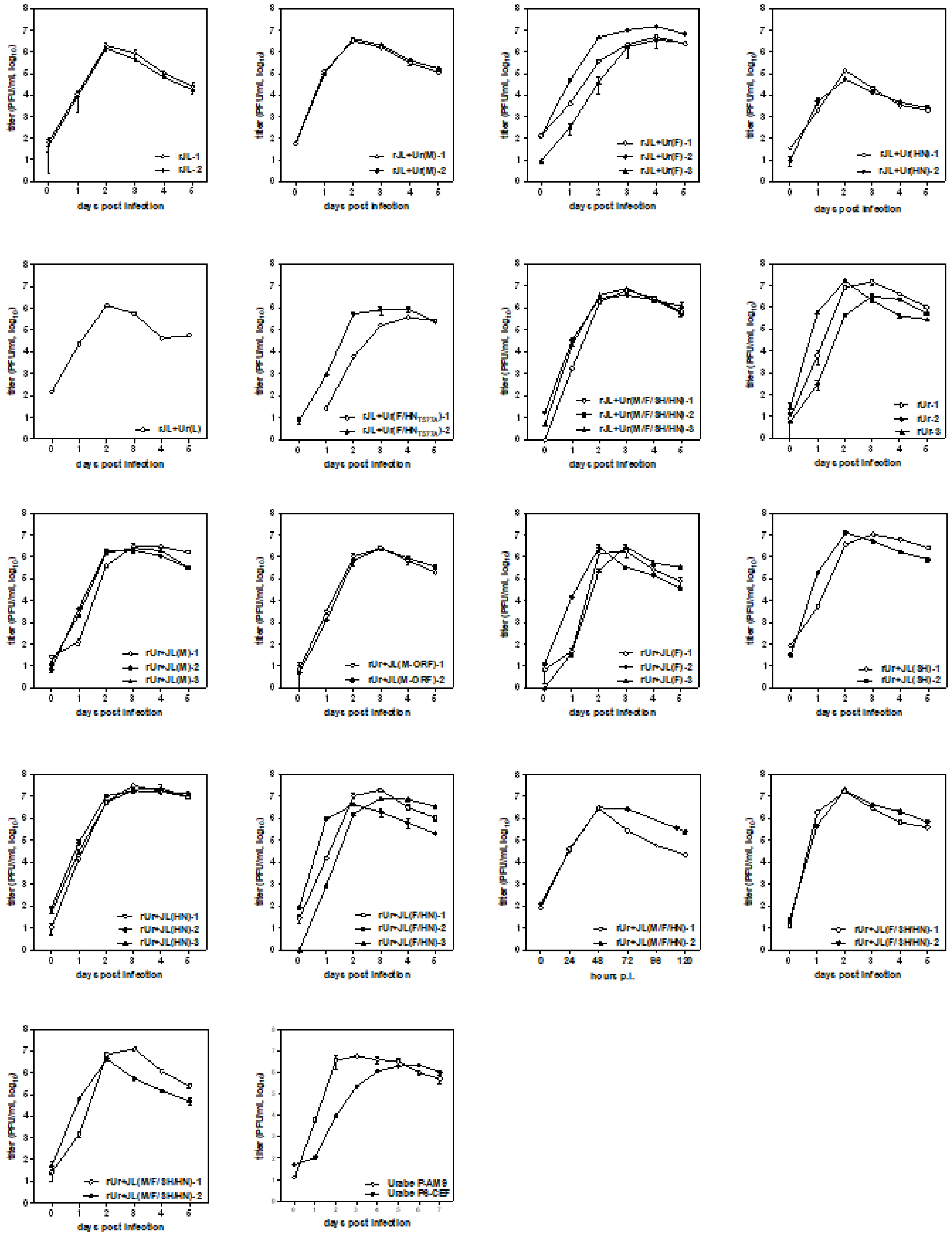

Supplemental Figure S1

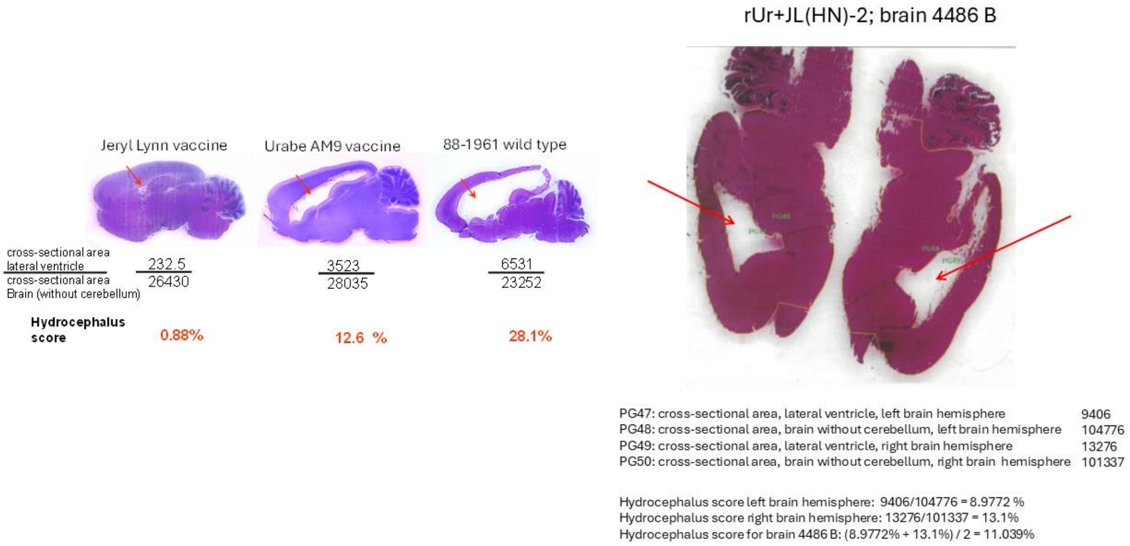

Supplemental Figure S2

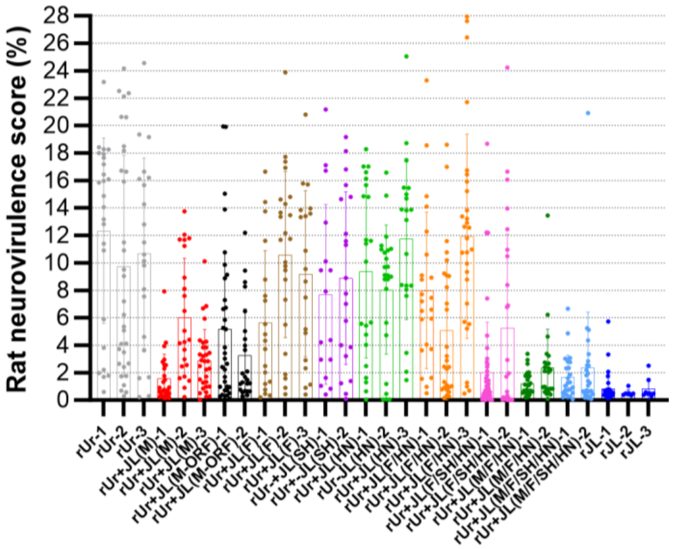

Supplemental Figure S3

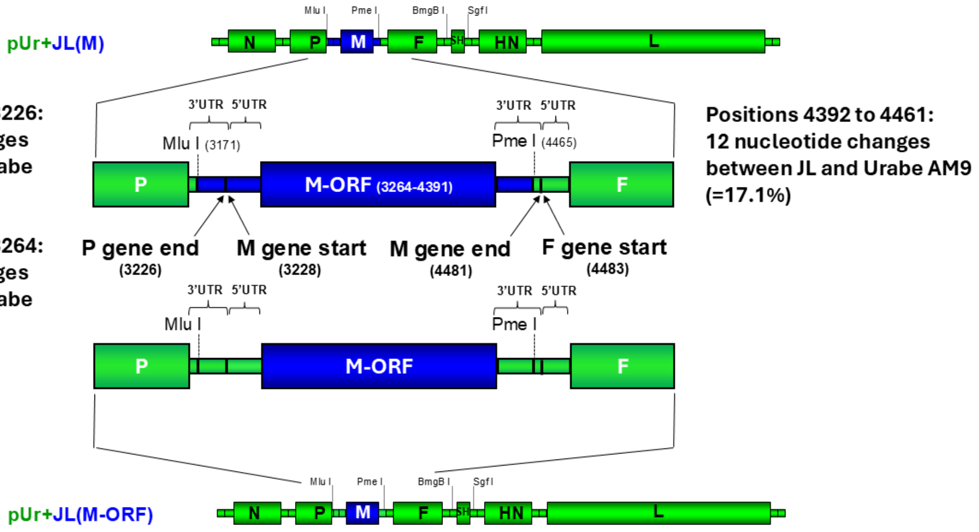

Supplemental Figure S4

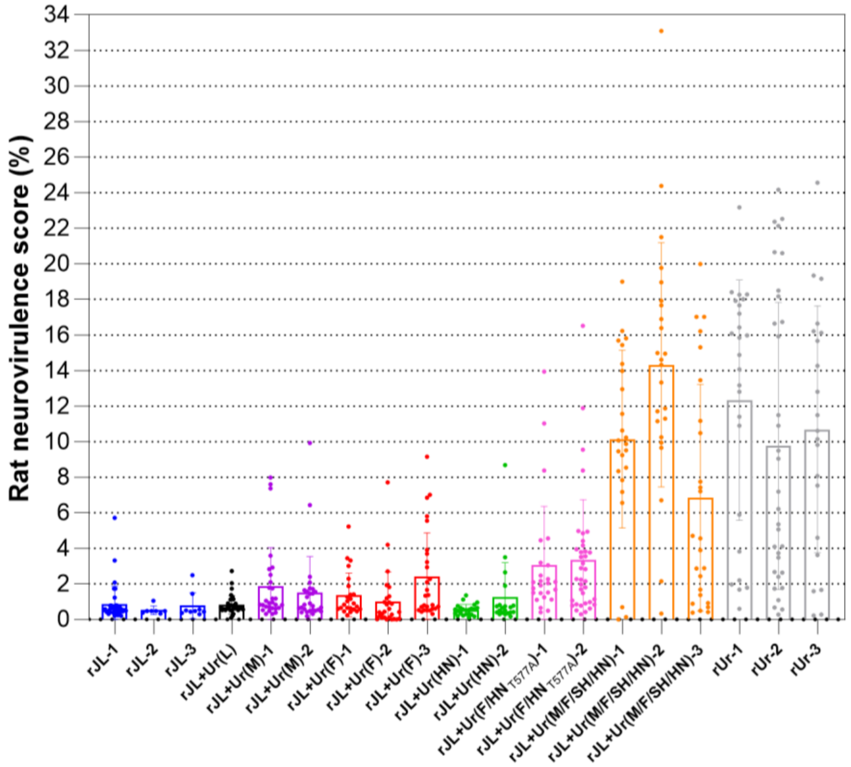

Supplemental Figure S5

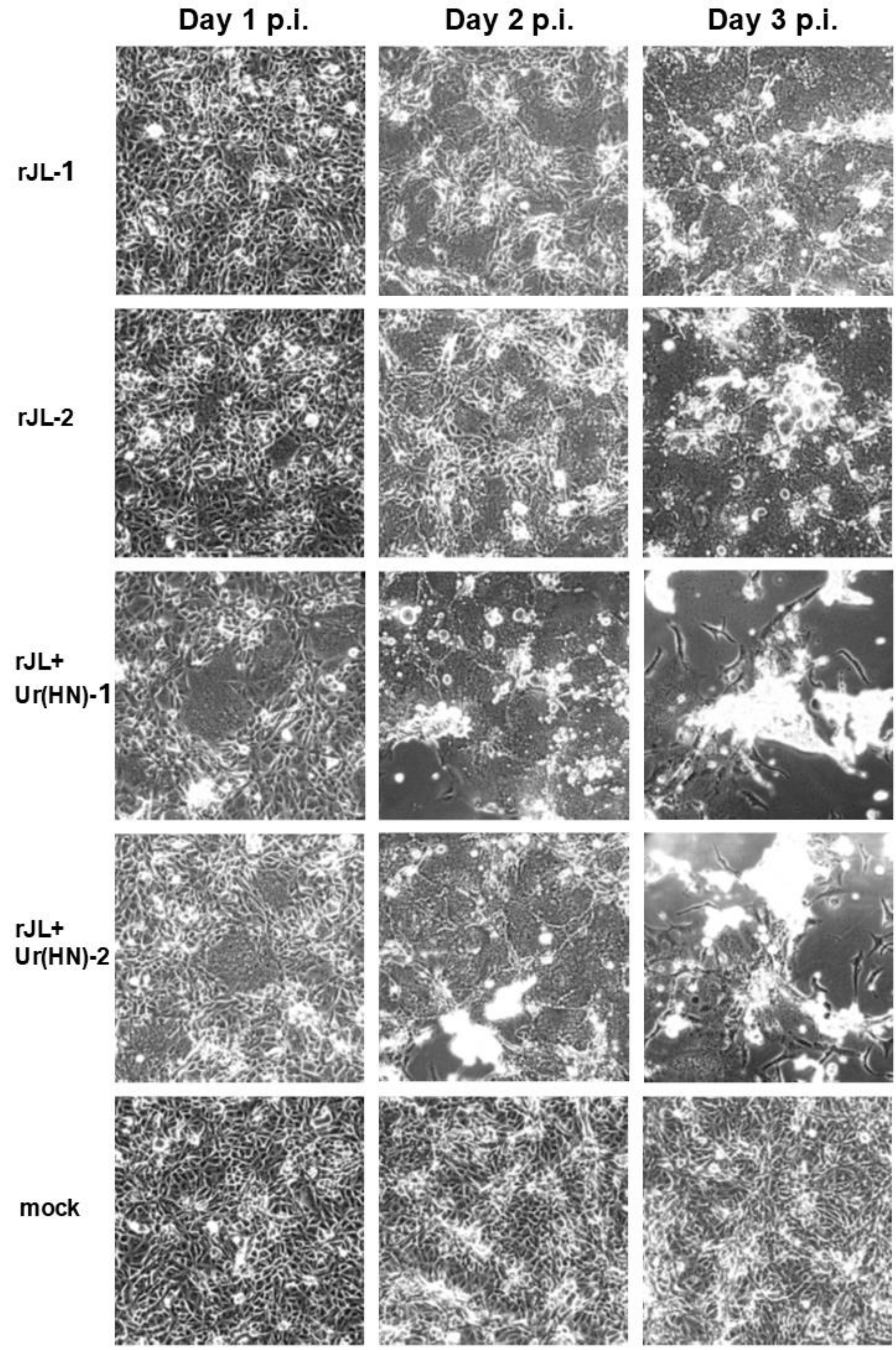

Supplemental Figure S6
