# Supplementary material for: A Chimeric Virus Approach Reveals the Matrix (M) Gene as a Critical Modulator of Mumps Virus Neurovirulence
Source: Vaccines (Basel). 2026 Sep 3;14(9):774. doi: 10.3390/vaccines14090774 (PMC13611789; doi:10.3390/vaccines14090774)
Supplement: Supplementary file 1 [file vaccines-14-00774-s001.zip › Supplemental Material and Methods-Generation of recombinant chimeric mumps virus cDNA constructs.pdf]

Supplemental Material and Methods

1. Generation of recombinant chimeric mumps virus cDNA constructs

1.1. Intermediate vectors used for the generation of recombinant chimeric mumps virus cDNA constructs:

The following cDNA constructs were used during the generation of the final cDNA constructs to rescue chimeric viruses employed for this study. Some plasmids are cited in section 1.2 below. Plasmids not cited in section 2.1. were additional intermediate constructs needed for construction of some of the intermediate plasmids listed below.

1.1.1. cDNA plasmid **pMuVUrabe$_{\mathbf{NheI}}$** coding for the full-length Urabe AM9 genome has been described in [38]. The HN gene in pMuVUrabe$_{NheI}$ is identical to that present in pURABE$_{HN\text{-}K335}$ except for nucleotide change C8005A resulting in amino acid change N464K. In addition, for cloning purposes, nucleotide change A8342G was introduced creating an *Nhe* I site that leads to amino acid change T577A in the Urabe AM9 HN protein. Of note, the *Nhe* I site is authentic in the JL genome encoded by the plasmid pMuV(MPBS).

1.1.2. cDNA plasmid **pMuVUrabe$_{\mathbf{NheI}}$(3'-NP-P-5'-JL)** has been described in [38]. The plasmid codes for the full-length Urabe genome except for the N and P genes as well as the genomic 3' and 5' ends which are derived from the JL virus strain.

1.1.3. TOPO-72-8236,1-5: RNA was extracted from Urabe P-AM9 (Original Urabe AM9 vaccine passaged twice in Vero cells [51] and *in vitro* transcribed using primer Mu7.2fwd [38]. A PCR fragment encompassing Urabe AM9 nucleotides 7200- 8236 bp (covering part of the HN gene) was generated using primers Mu7.2fwd and 8230 rev [38] and subcloned into plasmid pCR2.1 TOPO to yield **TOPO-72-8236,1-5.**

1.1.4. TopoJlHNL88: The plasmid contains the JL HN gene and the L gene from MuV strain 88-1961 [39].

1.1.5. TOPOSgfI/NheI [38]: The shuttle vector contains a PCR fragment encompassing nucleotides 6466 to 8343 of Urabe AM9. Briefly, the entire Urabe AM9 HN ORF (except for the last 18 nucleotides) and a region encompassing the HN 3' UTR, SH/HN intergenic (IG) region as well as part of the SH gene encompassing the SH gene 5' UTR was amplified using primers HNfwdSgfI (aagtc*gcgatcgc*tcatgcgagaacaag Sgf I site in italics) and HNFLNheIrev (ggtcaatctg*gctagc*ac aggtagaatttgg *Nhe*I site in italics) using plasmid **TOPO2.58-2.** (Refer to 1.1.9. below) as template. The fragment was subcloned into pCR2.1 TOPO to yield **TOPOSgfI/NheI**.

1.1.6. TOPOXhoI-SH-SgfI: The plasmid contains the Urabe SH ORF and parts of the flanking 5' and 3' UTRs. The gene was amplified using primers UrabshfwdbmgbI and UrabshrevsgfI [38] containing *BmgB* I (position 6246) and *Sgf* I (position

6461) restriction enzyme sites, respectively, and using as template plasmid TOPO2.58-3 (TOPO2.58-3 is identical to **TOPO2.58-2** (Refer 1.1.9. below) except for nucleotide change G8088A). The 227 bp PCR fragment was cloned into plasmid pCR2.1 TOPO to yield **TOPOXhoI-SH-SgfI** [38].

1.1.7. **Link TopoSgfIMluI:** To create a shuttle vector that has unique *Mlu*I and *Sgf*I sites, a linker was inserted into the unique *Xho*I site of shuttle vector **TOPO-SgfI/XhoIdelNhe** (containing the Urabe HN gene (with K335 and K464), and part of the Urabe L gene [38]). The following two oligonucleotides were hybridized to create the linker that was ligated into the *Xho*I site of TOPO-SgfI/XhoIdelNhe: 5’ tcgaggtttaaacacgcgtc 3’, and 5’ tcgagacgcgtgtttaaacc3’.

1.1.8. **TopoUrabeMluI-SgfI**: Contains the *Mlu*I-*Sgf*I fragment from Urabe AM9 coding for the Urabe M, F, and SH genes. The 3.3 kbp *Mlu*I/*Sgf*I fragment containing the Urabe M, F, and SH genes was excised from **pMuVUrabe$_{\mathbf{NheI}}$** (Refer to 1.1.1. above) and ligated into shuttle vector **LinkTopoSgfIMluI** (Refer to 1.1.7. above) restricted with *Sgf*I and *Mlu*I, to yield **TopoUrabeMluI-SgfI.**

1.1.9 **pTM1-HN-CEF$_{\mathbf{(K335/K464)}}$**: To generate a plasmid that allows expression of the Urabe HN gene that exhibits nucleotide “A” at positions 7616 and 8005 (A is predominant at these positions of heterogeneity in Urabe AM9 following six serial passages in chicken embryo fibroblast (CEF) cells [51] resulting in amino acids K335 and K464, respectively. RNA extracted from Urabe AM9 passaged six times in CEF cells was *in vitro* transcribed using primer PCR2-M (binding at genome position 3822 [38], and subsequently a PCR fragment encompassing nucleotides 5994-8571 was amplified und subcloned into pCRII- TOPO (Life Technologies / Invitrogen, now Thermo Fisher Scientific, Waltham, MA, USA) to give rise to plasmid **TOPO2.58-2.** A 1750 bp PCR fragment encompassing genomic positions 6614-8363, encoding the Urabe AM9 HN ORF was amplified by PCR with **TOPO2.58-2** as template and using primers HNUrNcoIfwd (ctcgaa*ccatgg*agccctcaaaactc; *Nco*I site is in italics) and HNUrXhoIrev (ca*ctcgag*actcaagtgatggtcaatctgg; *Xho*I site is in italics). The PCR fragment was subcloned into pCR2.1 TOPO (Life Technologies / Invitrogen, now Thermo Fisher Scientific, Waltham, MA, USA) to yield **pCR2.1-HNCEF**. An 1150 bp fragment encompassing nucleotides 7251 to 8363 of the HN gene was excised from pCR2.1-HNCEF using restriction sites *Nco*I and *Bam*HI (derived from pCR2.1-TOPO) and ligated into plasmid pTM1 (kindly provided by Bernard Moss (NIH, Bethesda, USA) (Moss, B., et al., *Nature* 1990, 348, 91-92)), restricted with restriction enzymes *Nco*I and *Bam*HI to create **pTM1-HNCEFdelNco**. Next, a 638 bp *Nco*I/*Nco*I fragment was excised from **pCR2.1-HNCEF** and ligated into **pTMI-HNCEFdelNcoI** restricted with *Nco*I to give rise to plasmid **pTM1-HN-CEF$_{\mathbf{(K335/K464)}}$**.

1.1.10. **pTM1-HN-WT$_{\mathbf{(E335/N464)}}$:** To generate a plasmid that allows expression of the Urabe AM9 HN gene that exhibits nucleotide “G” at position 7616 and nucleotide

"C" at position 8005 (predominant nucleotides at these two sites of heterogeneity in the original Urabe AM9 vaccine following two serial passages in Vero cells [51], therefore termed wildtype (WT) and translating in amino acids E335 and N464, respectively), plasmid **pTM1-HN-CEF$_{(K335/K464)}$** (refer to 1.1.9. above) was cut with restriction enzyme *Nde*I and the released 623 bp fragment (encompassing Urabe AM9 genomic nucleotides 7578-8202) was replaced with the corresponding *Nde*I fragment excised from shuttle vector **TOPO-72-8236,1-5** (refer to 1.1.3. above), to give rise to **pTMI-HN-WT$_{(E335/N464)}$.**

1.1.11. pCAGGS-MCS: Plasmid pCAGGS-MCS (kindly provided by Juan Carlos de la Torre, Scripps Research Institute, La Jolla, CA, USA), was generated by excision of a 92 bp fragment from plasmid **pCAGGS** (Niwa et al., *Gene* 1991, 108, 193-199) using restriction sites *EcoR* I and *Bgl* II and ligation of a 53 nt polylinker flanked by *Eco*RI (5' end) and *Bgl* II (3' end) restriction sites.

1.1.12. pCAGGS-HN-CEF$_{(K335/K464)}$: To generate a plasmid that allows expression of the Urabe AM9 HN$_{K335/K464}$ gene in the plasmid **pCAGGS-MCS** (refer to 1.1.11. above), the HNCEF gene was amplified by PCR using fwd primer HNUrSacIfwd (cat*gagctc*accatggagccctcaaaactc; *Sac*I site is in italics) and reverse primer HNUrXhoIrev (refer to 1.1.9 above) using plasmid **TOPO2.58-2** (refer to 1.1.9. above) as template. The PCR fragment was restricted with restriction enzymes *Sac*I and *Xho*I and ligated into **pCAGGS-MCS** restricted with *Sac*I and *Xho*I to give rise to **pCAGGS-HN-CEF$_{(K335/K464)}$**.

1.1.13. pCAGGS-HN-CEF$_{(K335/K464)}$-delSacI: In this plasmid, the *Sac*I site downstream of the *Eco*RI site in the polylinker region of **pCAGGS-HN-CEF$_{(K335/K464)}$** (Refer to 1.1.12. above) was removed by first linearizing the plasmid with *Sac*I followed by recession of the resulting 3' overhang of the *Sac*I -linearized DNA using the large (Klenow) fragment of DNA Polymerase I (New England Biolabs, Ipswich, MA, USA), thereby creating a 5' blunt end. The Urabe AM9 HN gene was then excised from the linearized plasmid using *Xho*I and ligated into **pCAGGS-HN-CEF$_{(K335/K464)}$** that was cut first with *Eco*RI, followed by treatment with the Klenow fragment to fill in the resulting 5' overhang, resulting in a 3' blunt end. The HN gene was removed from this construct with *Xho*I, to be replaced with the HN gene containing the 5' blunt end and the *Xho*I site at the 3' end.

1.1.14 pCAGGS-HN-WT$_{(E335/N464)}$-delSacI: The 871 bp fragment encompassing Urabe AM9 nucleotides 7387-8258 was excised from plasmid **pTM1-HN-WT$_{(E335/N464)}$** (Refer to 1.1.10. above) using *Bsm*BI and *Age*I restriction enzymes and ligated into **pCAGGS-HN-CEF$_{(K335/K464)}$-delSacI** (Refer to 1.1.13. above), from which the corresponding 871 bp fragment had been removed using *Bsm*BI and *Age*I restriction enzymes, to yield **pCAGGS-HN-WT$_{(E335/N464)}$-delSacI**.

1.1.15. pCAGGS-HN$_{(K335/N464)}$-delSacI. To allow expression of the HN$_{(K335/N464)}$ protein using the pCAGGS expression vector, the 373 bp fragment encompassing Urabe AM9 nucleotides 7885-8258 and displaying "C" at position 8005 (coding for

N464) and "G" at position 8015, was released from **pCAGGS-HN-WT(E335/N464)-delSacI** (Refer to 1.1.14. above) using *Pvu*II and *Age*I restriction enzymes and ligated into **pCAGGS-HN-CEF(K335/K464)-delSacI** (Refer to 1.1.13. above) from which the corresponding *Pvu*II / *Age*I 373 bp fragment had been removed. The resulting plasmid **pCAGGS-HN(K335/N464)-delSacI** exhibited an "A" at position 7616 (coding for K335) and "C" at position 8005 (coding for N464). For the sake of simplicity, the Urabe AM9 protein with K335 and N464 is referred to as Urabe AM9 "HN" gene throughout the manuscript, if not stated otherwise.

1.1.16. TOPO-XhoI-HN(K335/N464)delNhe-SgfI: The 626 bp fragment encompassing nucleotides 7576-8202 and including nucleotides A7616 (coding for HN K335) and C8005 (coding for HN N464) was excised from expression plasmid **pCAGGS-HN(K335/N464)-delSacI** (Refer to 1.1.15. above) using restriction enzyme *Nde* I, and ligated into HN shuttle vector **TOPO-XhoI-HNCEFdelNhe-SgfI** (Refer to 1.1.21 below under "Generation of cDNA plasmids pJL+Ur(HN-CEF) and pJL+Ur(F/HN-CEF)), from which the corresponding *Nde*I fragment was excised, to yield **TOPO-XhoI-HNK335/N464)delNhe-SgfI**.

1.1.17. pTMI-M (Urabe): The generation of this plasmid was described previously [38]. It contains Urabe AM9 sequences encompassing nucleotides 3163-4476 including the P 5'UTR, the P/M IG, the M 3' and 5' UTR, as well as the M gene. The UrabeAM9 specific sequence can be excised using *Mlu*I and *Pme*I restriction sites that were introduced into the primers used to amplify the Urabe AM9 sequence.

1.1.18. Generation of cDNA plasmid pJL+Ur(HN-CEFT577A): The generation of this plasmid was previously described in [38]. In the previous study, the plasmid was used as an intermediate step for generation of the full-length clone of Urabe AM9. Briefly, the entire Urabe AM9 HN ORF (except for the last 18 nucleotides) and a region encompassing the HN 3' UTR, SH/HN intergenic (IG) region as well as part of the SH gene encompassing the SH gene 5' UTR was amplified using primers HNfwdSgfI (aag tc*gcgatcgc*tcatgcgagaacaag Sgf I site in italics) and HNFLNheIrev (ggtcaatctg*gctagc*ac aggtagaatttgg *Nhe*I site in italics) using plasmid **TOPO2.58-2.** (Refer to 1.1.9.above) as template. The fragment was subcloned into pCR2.1 TOPO to yield **TOPOSgfI/NheI** (Also described in 1.1.5 above). Sequencing of the subcloned fragment (encompassing nucleotides 6466-8343 of Urabe AM9) confirmed identity to the published sequence of Urabe AM9 (NCBI Genbank Acc.no. AF314559) except for the intentionally introduced *Sgf*I and *Nhe*I sites at positions 6466 and 8338, respectively. The *Nhe*I site was transiently introduced for cloning purposes taking advantage of a unique *Nhe*I site present in the HN gene of JL in the cDNA plasmid pMuV(MPBS), and was later removed again by site-directed mutagenesis (Refer to 1.1.21. below). The nucleotide at position 7409, referred to as "R" in NCBI Genbank Acc. no. AF314559 was identified as "G" in agreement with [47]. The 1877 bp *Sgf*I/*Nhe*I fragment from pMuV(MPBS) was replaced with the corresponding subcloned

*Sgf*I/*Nhe*I Urabe AM9 fragment to give rise to pJL+Ur(HN-CEF$_{T577A}$). Of note, the plasmid was termed pMuVJL(HN$_{NheI}$-Urabe) in [38].

1.1.19. Generation of cDNA plasmid pJL+Ur(F/HN-CEF$_{T577A}$): The 1790 bp *Pme*I/*Bmg*BI fragment from **pJL+Ur(HN-CEF$_{T577A}$)** (Refer to 1.1.18. above) was replaced with the corresponding *Pme*I/*Bmg*BI fragment from **pTM1-Fwt** (Refer to 1.2.1. below) to give rise to **pJL+Ur(F/HN-CEF$_{T577A}$)**.

1.1.20. Generation of cDNA plasmid pJL+Ur(F/SH/HN-CEF$_{T577A}$): The *Bmg*BI/*Sgf*I fragment containing the SH ORF and parts of its 5' and 3' UTRs was excised from shuttle vector **TOPOXho-SH-SgfI**. (Refer to 1.1.6. and [38] and ligated into **pJL+Ur(F/HN-CEF$_{T577A}$)** (Refer to 1.1.19. above), from which the corresponding *Bmg*BI/*Sgf*I fragment was removed, to yield **pJL+Ur(F/SH/HN-CEF$_{T577A}$)**.

1.1.21. Generation of cDNA plasmids pJL+Ur(HN-CEF) and pJL+Ur(F/HN-CEF): First, the *Sgf*I/*Xho*I fragment from **pJL+Ur(F/HN-CEF$_{T577A}$)** (Refer to 1.1.19. above) encompassing nucleotides 6461 to 8881, and including the HN gene, and part of the L gene, was subcloned into **TOPOXhoI-SH-SgfI** (Refer to 1.1.6. above and [38]), from which the *Xho*I/*Sgf*I fragment had been removed, to yield **TOPO-XhoI-HNCEF$_{T577A}$-SgfI**. The *Nhe* I site at position 8338 (that was introduced into the Urabe AM9 HN gene by nucleotide change A8342G resulting in aa change T577A) was removed by site-directed mutagenesis (introducing nucleotide change G8342A) using overlap extension PCR [55]. Two overlapping PCR fragments were generated using primer combinations HNfwd7893/HNendnhedelrev and HNendnhedelfwd/9082rev [38], with **pJL+Ur(F/HN-CEF$_{T577}$)** as the template. The resulting PCR fragment encompassing nucleotides 7893-9082 was cloned into pCR2.1 TOPO. A 966 bp fragment encompassing Urabe AM9 nucleotides 7915-8881 including the G8342A nucleotide change that destroyed the *Nhe* I site and restored the Urabe HN gene sequence), was excised from the subcloned PCR fragment using restriction enzymes *Eco*RV and *Xho*I and ligated into **TOPO-XhoI-HNCEF$_{T577A}$-SgfI** from which the corresponding *Eco*RV/*Xho* I fragment had been removed, to yield **TOPO-XhoI-HNCEFdelNhe-SgfI**. Finally, the *Sgf*I/*Xho*I fragment encompassing nucleotides 6461 to 8881was excised from **TOPO-XhoI-HNCEFdelNhe-SgfI** and ligated into **pJL+Ur(F/HN-CEF$_{T577A}$)** from which the corresponding *Sgf*I/*Xho*I fragment had been removed, to yield **pJL+Ur(F/HN-CEF)**. The same *Sgf*I/*Xho*I fragment excised from **TOPO-XhoI-HNCEFdelNhe-SgfI** and containing A8342 also was ligated into **pJL+Ur(HN-CEF$_{T577A}$)** (Refer to 1.1.18. above) from which the corresponding *Sgf*I/*Xho*I fragment had been removed, to yield **pJL+Ur(HN-CEF)**.

1.1.22. Generation of cDNA plasmid pJL+Ur(M/F/SH/HN-CEF$_{T577A}$). This cDNA plasmid was used as intermediate to generate plasmid **pJL+Ur(M/F/SH/HN)** (Refer to 1.2.3. below). The 1.3 kbp *Mlu*I/*Pme*I fragment (encompassing Urabe AM9 nucleotides 3163-3840 including the Urabe AM9 M ORF, the P gene 5'UTR, the P/M intergenic region, the M 3' -and -5'UTRs) was excised from plasmid **pTM1-M** (Refer to 1.1.17. above), and ligated into **pJL+Ur(F/SH/HN-CEF$_{T577A}$**

(Refer to 1.1.20. above), from which the corresponding *Mlu*I/*Pme*I fragment (including the JL M gene) had been removed, to yield plasmid **pJL+Ur(M/F/SH/HN-CEF$_{T577A}$)**. Of note, generation of this plasmid had been described previously in [38] and was referred to as plasmid pMuVJL(M-F-SH-HN$_{NheI}$ Urabe).

1.2. Generation of the cDNA plasmids that were used to rescue the recombinant chimeric mumps viruses shown in Figure 2.

1.2.1. Generation of cDNA plasmid pJL+Ur(F): The Urabe AM9 F gene together with the M/F IG region, the F 3'UTR, the F 5'UTR, the F/SH IG region as well as a part of the SH 3'UTR were first assembled with two fragments in plasmid pTM1. Fragment 1 was amplified with primers F-PmeIfwd and primer 38039 [40] using as template a subcloned 1,52 kbp Urabe AM9 cDNA [51]. The obtained 0.9 kbp PCR fragment was subcloned and a 0.8 kbp fragment encompassing Urabe AM9 sequences 4451-5248 was ligated into pTM1 via *Kpn*I and *Pst*I to create **pTM1-F1**. Fragment II was amplified with primers Fmidfwd and BmgBIrev [38] using as template a subcloned 1,55 kbp Urabe AM9 cDNA [51]. The 1.2 kbp PCR fragment was subcloned and contained- in addition to the newly created *Bmg*BI site- two silent mutations (G5460A and C5782T) as well as the pos 5653 A→G nucleotide change (resulting in aa change T370A in the F protein), that was found to be predominant in Urabe AM9 following passaging in CEF cells [51]. A 1015 bp fragment encompassing Urabe AM9 sequences 5243-6251 was ligated into pTM1-F1 via *Pst*I and *Xho*I to give rise to **pTM1-Fmut**. Nucleotide position 5653 was subsequently reversed by site-directed mutagenesis from "g" to "a" employing overlap extension PCR [55] using two overlapping PCR fragments that were generated using primers Fmidfwd, Fur5653rev, Furwt5653fwd and FurXhorev [38]. Following subcloning, a 523 bp *Nsi*I fragment (pos 5393-5915) containing nucleotide A5653 was removed from the subclone and ligated into *Nsi*I restricted plasmid **pTM1-Fmut** to give rise to **pTM1-Fwt**. Next, the 1790 bp *Pme*I/*Bmg*BI fragment containing the JL F gene was excised from pMuV(MPBS) and replaced with the *Pme*I/*Bmg*BI fragment excised from **pTMI-Fwt** containing the Urabe AM9 F gene, yielding cDNA plasmid **pJL+Ur(F)** (Figure 2).

1.2.2. Generation of cDNA plasmid pJL+Ur(F/HN$_{T577A}$): First, the *Nde*I fragment encompassing nucleotides 7576-8202 (including A7616 and C8005, coding for K335 and N464 in the HN protein), was excised from expression plasmid **pCAGGS-HN$_{(K335/N464)}$-delSacI** (Refer to 1.1.15. above) and ligated into HN shuttle vector **TOPOSgfI/NheI (**Refer to 1.1.5. above) from which the *Nde*I fragment (containing A7616 and A8005, coding for K335 and K464 in the HN protein) had been removed, to yield **TOPOSgfI/NheI-HN$_{(K335/N464)}$.** Next, the *Sgf*I/*Nhe*I fragment from **TOPOSgfI/NheI-HN$_{(K335/N464)}$** was ligated into cDNA plasmid **pJL+Ur(F)** (Refer to 1.2.1. above) from which the JL *Sgf*I/*Nhe*I fragment had been removed, to yield **pJL+Ur(F/HN$_{T577A)}$** (Figure 2).

1.2.3. Generation of cDNA plasmid pJL+Ur(M/F/SH/HN): To construct a full-length cDNA clone in which the JL M, F, SH, and HN genes were replaced with the corresponding genes from Urabe AM9 (pJL+Ur(M/F/SH/HN), the 2422 bp fragment encompassing nucleotides 6466-8883 (containing the Urabe AM9 HN gene and part of the JL L gene) was removed from shuttle vector **TOPO-XhoI-HN($_{K335/N464}$)delNhe-SgfI** (Refer to 1.1.16. above) using restriction enzyme sites *Xho*I and *Sgf*I, and ligated into plasmid **pJL+Ur(M/F/SH/HN-CEF$_{T577A}$)** (Refer to 1.1.22. above) from which the corresponding *Sgf*I/*Xho*I fragment had been removed, to yield plasmid **pJL+Ur(M/F/SH/HN)** (Figure 2).

1.2.4. Generation of cDNA plasmid pJL+Ur(HN): To generate cDNA clone **pJL+Ur(HN)** (Figure 2), the 2422 bp *Sgf*I/*Xho*I fragment encompassing nucleotides 6466-8883 (containing the Urabe AM9 HN$_{(K335/N464)}$ gene and part of the JL L gene) was removed from plasmid **JL+Ur(M/F/SH/HN**) (Refer to 1.2.3. above) and ligated into pJL (= pMuV(MPBS)) from which the corresponding SgfI/XhoI fragment had been removed.

1.2.5. Generation of cDNA plasmid pJL+Ur(M): To construct a full-length cDNA clone in which the JL M gene has been replaced with the Urabe AM9 M gene (**pJL+Ur(M)**)(Figure 2), the Urabe AM9 M gene was excised from **pTMI-M** (Refer to 1.1.17. above)) using *Mlu*I and *Pme*I restriction enzymes and ligated into plasmid **pMuV(MPBS)** from which the JL M gene was removed using *Mlu*I and *Pme*I restriction enzymes).

1.2.6. Generation of cDNA plasmid pJL+Ur(L): To construct a full-length cDNA clone in which the JL L gene had been replaced with the Urabe AM9 L gene (**pJL+Ur(L)**, (Figure 2), the Urabe AM9 specific 6.89 kbp *Nhe*I/*Swa*I fragment (encompassing nucleotides 8338-15225 coding for the Urabe L gene together with the Urabe AM9 5'UTR, the HN/L intergenic region as well as the Urabe AM9 L 3'UTR) was excised from plasmid **pMuVUrabe$_{NheI}$(3'-NP-P-5'-JL)** (Refer to 1.1.2. above, and [38]) and ligated into **pMuV(MPBS)** from which the JL L gene had been removed using restriction enzymes *Nhe*I and *Swa*I.

1.2.7. Generation of cDNA plasmid pUr+JL(HN): The JL HN gene was excised from shuttle vector **Topo JLHNL88** (Refer to 1.1.4. above) using the enzymes *Sgf*I and *Nhe*I, and this fragment was ligated into **pMuVUrabe$_{NheI}$** (Refer to 1.1.1. above and [38]), from which the corresponding *Sgf*I/*Nhe*I fragment had been removed, to yield **pUr+JL(HN)** (Figure 2).

1.2.8. Generation of cDNA plasmid pUr+JL(SH): To construct a full-length clone in which the Urabe AM9 SH gene had been replaced with the JL SH gene, the JL SH gene was excised from **pMuV(MPBS)** using restriction enzymes *Bmg*BI and *Sgf*I and cloned into shuttle vector **TopoUrabeMluI-SgfI** (Refer to 1.1.8. above) restricted with *Bmg*BI and *Sgf*I to create **TopoUrabeMluI-SgfIJLSH**. The *Pme*I-*Sgf*I fragment of **TopoUrabeMluI-SgfIJLSH**, that contains the Ur F gene and the JL SH gene, was removed from the shuttle vector and was ligated into

pURABE$_{\mathbf{HN\text{-}K335}}$ from which the corresponding *Bmg*BI/*Sgf*I fragment had been removed to yield **pUr+JL(SH)** (Figure 2).

1.2.9. Generation of cDNA plasmid pUr+JL(F): The generation of this plasmid was already described previously in [58]. Briefly, to construct a full-length clone in which the Urabe AM9 F gene had been replaced with the JL F gene, the JL F gene fragment was first excised from pMuV(MPBS) using restriction enzymes *Pme*I and *Bmg*BI and subcloned into the **TopoUrabeMluI-SgfI** shuttle vector (Refer to 1.1.8. above) restricted with *Pme*I and *Bmg*BI, to create **TopoUrabeMluI-SgfIJLF**. The *Pme*I-*Sgf*I fragment containing the JL F gene and the Urabe AM9 SH gene was excised from **TopoUrabeMluI-SgfIJLF** using restriction enzymes *Pme*I and *Sgf*I. This fragment was ligated into the **pURABE$_{\mathbf{HN\text{-}K335}}$** backbone from which the corresponding *Pme*I/*Sgf*I fragment had been excised, to yield **pUr+JL(F)** (Figure 2).

1.2.10 Generation of cDNA plasmid pUr+JL(M): To construct a full-length clone in which the Urabe AM9 M gene had been replaced with the JL M gene (**pUr+JL(M)**), the JL M gene was removed from **pMuV(MPBS)** using restriction enzymes *Mlu*I and *Pme*I, and ligated into the pURABE$_{HN\text{-}K335}$ backbone restricted with *Mlu*I and *Pme*I, to yield **pUr+JL(M)** (Figure 2).

1.2.11. Generation of cDNA plasmid pUr+JL(M-ORF): To construct a full-length clone, in which only the Urabe AM9 M-ORF, but not the flanking untranslated regions of the Urabe AM9 M-ORF was replaced with the corresponding JL M-ORF, the following DNA fragment was synthesized and subcloned into plasmid pEX-K4 by Eurofins Genomics (Huntsville, AL, USA):

5’CCAG***ACGCGT***GGGAAAATCCACAAACTGAAAGCCACAATGATTCCCT
GTTAAATAAAAAAT**AAGCACGAA**CACAAGTCCAATCCAACCATAGCAG
CAatggcaggatcacagatcaaaattcctcttccaaagccccccgattcagactctcaaagactaaatgccttccctgt
catcatggctcaagaaggcaaaggacgactccttagacaaatcaggcttaggaaaatattatcaggggatccgtctga
tcagcaaattacatttgtgaatacatatggattcatccgtgccactccagaaacatccgagttcatctctgaatcatcacaa
caaaaggtaactcctgtagtgacagcgtgcatgctgtcctttggtgccggaccagtgctagaagatccacaacatatgc
tcaaggctcttgatcagacagacattagggttcggaaaacagcaagtgataaagagcagatcttattcgagatcaacc
gcatccccaatctattcaggcattatcaaatatctgcggaccatctgattcaggccagctccgataaatatgtcaaatcac
cagcaaaattgattgcaggagtaaattacatctactgtgttacattcttatctgtgacagtttgttctgcctcactcaagtttc
gagttgcgcgcccattgcttgctgcacggtccagattagtaagagcagttcagatggaaattttgcttcgggtaacttgc
aaaaaagattctcaaatggcaaagagcatgttaaatgaccctgatggagaagggtgcattgcatccgtgtggttccacc
tatgtaatctgtgcaaaggcagaaataaacttagaagttacgatgaaaattattttgcttctaagtgccgtaagatgaatct
gacagtcagcataggagatatgtggggaccaaccattctagtccatgcaggcggtcacattccgacaactgcaaaac
ctttttcaactcaagaggctgggtctgccacccaatccaccaatcatcaccatcgttggcgaagaccctatggtcatct
gggtgtgaaatcaaggctgccagtgctattctccagggttcagactatgcatcacttgcaaagactgatgacataatata
ttcgaagataaaagtcgataaagacgcggccaactacaaaggagtatcctggagtccattcaggaagtctgcctcaat
gagaaacctatgaGAATTTCATCTATTCCCCCTGATGCCTCCAGGAGAATCAAC
AATCAGTCCGATTTTACCGGTGGTAACTT***GTTTAAAC***TTATAG 3’.

The flanking 5’ *Mlu*I and 3’ *Pme*I restriction sites located at genomic positions 3171 and 4465, respectively, are both highlighted in bold and italics. The M gene start sequence at position 3228 is highlighted in bold letters. The JL M-ORF specific sequence encompassing nucleotides 3264-4391 is indicated in lower case letters. All nucleotides in upper case letters are Urabe AM9 specific. The 1298 bp DNA fragment encompassing genomic nucleotides 3171- 4469, encoding the JL M-ORF and flanking 3’ and 5’ UTRs from MuV Urabe AM9, was excised from the plasmid using restriction enzymes *Mlu*I and *Pme*I, and ligated into the **pURABE$_{\mathbf{HN\text{-}K335}}$** backbone from which the corresponding *Mlu*I/*Pme*I fragment had been removed, to yield **pUr+JL(M-ORF)** (Figure 2 and Figure S4)**.**

1.2.12. Generation of cDNA plasmid pUr+JL(F/HN): The generation of this plasmid was already described previously in [58]. Briefly, **pUr+JL(HN)** (Refer to 1.2.4.) was used to construct a full-length clone in which the Urabe AM9 HN and F genes had been replaced with the JL HN and F genes (pUr+JL(F/HN). The *Pme*I-*Sgf*I fragment containing the JL F and the Urabe AM9 SH gene was excised from shuttle vector **TopoUrabeMluI-SgfIJLF** (Refer to 1.2.9. above, description of cDNA plasmid **pUr+JL(F)**), using the *Pme*I and *Sgf*I restriction enzymes. This fragment was ligated into the **pUr+JL(HN)** backbone from which the corresponding *Pme*I/*Sgf*I fragment had been removed, to yield **pUr+JL(F/HN).**

1.2.13. Generation of cDNA plasmid pUr+JL(M/F/SH/HN): To construct a full-length clone in which the Urabe AM9 M, F, SH, and HN genes had been replaced with the JL M, F, SH and HN genes (**pUr+JL(M/F/SH/HN)**), the M, F, SH and HN genes were removed as a cassette from pMuV(MPBS) using the restriction enzymes *Mlu*I and *Nhe*I. This fragment was ligated into **pMuVUrabe$_{\mathbf{NheI}}$** (Refer to 1.1.1., above), from which the corresponding *Mlu*I/*Nhe*I-fragment had been removed.

1.2.14. Generation of cDNA plasmid pUr+ JL(F/SH/HN): To construct a full-length clone in which the Urabe AM9 F, SH and HN genes had been replaced with the JL F, SH, and HN genes (**pUr+JL(F/SH/HN)**), the Urabe AM9 M gene was excised from **pTM1-M (Urabe)** (Refer to 1.1.17. above) using *Mlu*I and *Pme*I restriction enzymes and ligated into **pUr+JL(M/F/SH/HN)** from which the JL M gene had been removed with the *Mlu*I and *Pme*I restriction enzymes.

1.2.15. Generation of cDNA plasmid pUr+JL(M/F/HN): To construct a full-length clone in which the Urabe AM9 M, F and HN genes had been replaced with the JL M, F, and HN genes (**pUr+JL(M/F/HN)**), the JL M gene was removed from pMuV(MPBS) using restriction enzymes *Mlu*I and *Pme*I and ligated into **pUr+JL(F/HN)** (described above) restricted with *Mlu*I and *Pme*I.

For all ligation reactions, the Quick Ligase kit (NEB Biolabs, Ipswich, MA, USA) was employed. Ligation reactions were transformed into Subcloning efficiency DH5α competent cells (Invitrogen/ThermoFisher Scientific). DNA fragments that were ligated into the full-length

JL or Urabe AM9 cDNA plasmids used DH5α cells that were rendered competent using Z-competent *E.coli* transformation kit (G-Biosciences, St. Louis, MO, USA) to achieve high transformation efficiency.
