# Supplementary material for: A Chimeric Virus Approach Reveals the Matrix (M) Gene as a Critical Modulator of Mumps Virus Neurovirulence
Source: Vaccines (Basel). 2026 Sep 3;14(9):774. doi: 10.3390/vaccines14090774 (PMC13611789; doi:10.3390/vaccines14090774)
Supplement: Supplementary file 1 [file vaccines-14-00774-s001.zip › Supplemental Table S1.pdf]

Table S1: Allelic variants ≥ 10% identified in rescued viruses.

Virus	Gene	Nucleotide change (percent)	Amino acid substitution (Protein), nature of change	Physico-chemical distance (D)[b]	Conser-vation among NNS viruses[c]
rJL-1[a]	-	-	-	-	-
rJL-2	-	-	-	-	-
rJL-3	L	A14269G (18%)	s	-	-
		A14397G (20%)	N1987S (L), cs	46	nc
		A14399G (20%)	N1988D (L), rs	23	c
rJL+Ur(M)-1	-	-	-	-	-
rJL+Ur(M)-2	-	-	-	-	-
rJL+Ur(F)-1	-	-	-	-	-
rJL+Ur(F)-2	M	G3437A (35%)	s	-	-
rJL+Ur(F)-3	HN	C7529T (11%)	P306S, rs	74	-
	L	T13210G (11%)	s (S1591)	-	-
rJL+Ur(HN)-1	L	T14250A (27%)	L1938Q (L), rs	113	nc
	V/P/I	G2619A (13%)	A215T (P), rs	58	-
			C214Y (V), rs	194	-
	L	C14113T (11%)	s	-	-
rJL+Ur(HN)-2	HN	A7653T (49%)	Y347F (HN), cs	22	-
	L	A14923C (24%)	L2162F (L), rs	22	nc
rJL+Ur(L)	HN5UTR	A6546C (16%)	-	-	-
	L	G8552T (17%)	D39F (L)[d], rs	177	nc
	L	A8553T (17%)			
	L	C14358T (20%)	S1973L (L) rs	145	nc
rJL+Ur(F/HN$_{T577A}$)-1	HN	T8222C (21%)	F537L (HN), rs	22	-
	L	G11733T (14%)	R1099M (L), rs	91	Bl IV, nc
rJL+Ur(F/HN$_{T577A}$)-2	SH	C6721A (28%)	P2T (SH), rs	38	-
	L	A9040G (17%)	s	-	-
rJL+Ur(M/F/SH/HN)-1	-	-	-	-	-
rJL+Ur(M/F/SH/HN)-2	**L**	**T11936G (53%)**	**L1167V (L) cs**	**32**	**Bl V, c**
rJL+Ur(M/F/SH/HN)-3	**L**	**T12356G (62%)**	**L1307V (L) cs**	**32**	**Bl V, c**
rUr+JL(M)-1	L	T9051C (29%)	V205A (L) cs	64	nc
	L	C10058T (26%)	L541F (L), rs	22	Bl II, c
	L	A9001G (21%)	s	-	-
	L	C14256T (20%)	T1940M (L), rs	81	nc
	L	C8842A (14%)	s	-	-
	L	G12732T (13%)	G1432V (L), cs	109	nc
rUr+JL(M)-2	L	C13250A (16%)	Q1605K (L), rs	53	nc
rUr+JL(M)-3	L	G9044T (21%)	D203Y (L), rs	160	nc
	V/P/I	G2823A (18%)	G282R (P), rs	125	-
	L	A12545G (10%)	s	-	-
rUr+JL(M-ORF)-1	L	A14735G (14%)	I2100V (L) cs	29	nc
	L	G8972A (10%)	V179I (L) cs	29	nc
rUr+JL(M-ORF)-2	-	-	-	-	-
rUr+JL(F)-1	L	A11911T (14%)	L1158F (L), rs	22	BL V, c
	N	A1184T (22%)	N347I (N), rs	149	-
	**F-GE**	**Ins A6203 (73%)**	**-**	-	-
rUr+JL(F)-2	F-GE	Ins A6203 (20%)	-	-	-
rUr+JL(F)-3	**L**	**T9110C (59%)**	**Y225H (L), rs**	**83**	**nc**
	**L**	**C10068A (58%)**	**P544H (L), rs**	**77**	**Bl II, c**
	P	C3050T (34%)	s	-	-

	N	G1731A (10%)	G529E (N), rs	98	**-**
	M3UTR	C4398T (10%)	-	-	**-**
rUr+JL(SH)-1	L	G14297A (23%)	A1954T (L), rs	58	nc
	L	G10531T (13%)	L698F (L), rs	22	Bl III, c
	F-GE	Ins A6203 (37%)	-	-	-
rUr+JL(SH)-2	V/P/I	G2823A (38%)	G282R (P), rs	125	-
	L	C10509T (18%)	S691L (L), rs	145	Bl III, c
rUr+JL(HN)-1	V/P/I	G2143A (14%)	s	-	-
	L	A10000C (12%)	s	-	-
	L	G10913A (16%)	E826K (L), rs	56	Bl III, c
	L	C14172T (13%)	A1912V (L), cs	64	nc
	HN	A7239G (10%)	N209S (HN), rs	46	-
	F-GE	Ins A6203 (11%)	-	-	-
rUr+JL(HN)-2	F	G4879T (10%)	A112S (F), rs	99	-
	F	T5091C (39%)	s	-	-
	L	T10571C (35%)	Y712H (L), rs	83	Bl III, c
	F-GE	Ins A6203 (22%)	-	-	-
rUr+JL(HN)-3	**L**	**C9636A (53%)**	**T400N (L), rs**	**69**	**Bl I, nc**
	**L**	**C9637G (51%)**	**T400L (L), rs**	**92**	**Bl I, nc**
	**L**	**C9636A+C9637G**	**T400K (L), rs**	**78**	**Bl I. nc**
	F-GE	Ins A6203 (24%)	-	-	-
rUr+JL(F/HN)-1	**F-GE**	**Ins A6203 (60%)**	-	-	-
	L-GE	A15359C (23%)	-	-	-
rUr+JL(F/HN)-2	N5UTR	T130A (30%)	-	-	-
	V/P/I	A2710G (29%)	Q244R (P), rs	76	-
	L	G11669T (29%)	R1078I (L), rs	97	Bl IV, c
	HN	T7402G (13%)	s	-	-
	F-GE	Ins A6203 (22%)	-	-	-
rUr+JL(F/HN)-3	L	A9741C (20%)	E435A (L), rs	107	Bl I, c
	L	G8947T (10%)	W170C (L), rs	215	nc
	F-GE	Ins A6203 (22%)	-	-	-
	**M-GE**	**Ins A4475 (59%)**	**-**	**-**	-
rUr+JL(F/SH/HN)-1	L	G9587T (26%)	E384STOP (L)	-	Bl I, nc
	L	C12642T (29%)	P1402L (L), cs	98	Bl V, nc
	L	T11555C (14%)	F1040L (L), rs	22	Bl IV, nc
	F-GE	Ins A6203 (12%)	-	-	-
rUr+JL(F/SH/HN)-2	L	A11149C (22%)	R904S (L), rs	110	nc
	L	C9114T (14%)	S226F (L), rs	155	nc
	L	A9002C (13%)	M189L (L), cs	15	c
	HN-GE	Ins A8422 (12%)	-	-	-
rUr+JL(M/F/HN)-1	F-GE	Ins A6203 (19%)	-	-	-
rUr+JL(M/F/HN)-2	**L**	**C8660T (61%)**	**P75S (L), rs**	**74**	**nc**
	L	C8522T (17%)	H29Y (L), rs	83	nc
	M	A3307G (14%)	D15G (M), rs	94	
rUr+JL(M/F/SH/HN)-1	**L**	**A9079G (77%)**	**s**	**-**	**-**
	**L**	**A9086G (74%)**	**I217V (L), cs**	**29**	**nc**
	**L**	**A9297G (78%)**	**K287R (L), cs**	**26**	**Bl I, nc**
rUr+JL(M/F/SH/HN)-2	L	G9901T (20%)	W488C (L), rs	215	c
	L	G10513A (21%)	M692I (L), cs	10	Bl III, c
	F-GE	Ins A6203 (14%)	-	-	-
rUr-1	F	A4609G (17%)	I22V (F), cs	29	-
	L	G11333A (11%)	A966T (L), rs	58	Bl IV, c
	L	C9851A (10%)	L472I (L), cs	5	c
	F-GE	Ins A6203 (15%)	-	-	-
rUr-2	F-GE	Ins A6203 (27%)	-	-	-

rUr-3	V/P/I<br>L	G2823A (47%)<br>**C14358T (61%)**	G282R (P), rs<br>**S1974L (L), rs**	125<br>145	-<br>nc

[a] virus rJL-1 was sequenced using Sanger sequencing only (nucleotides 24-15362); all other viruses were sequenced by HTS

[b] distance (D) according to R. Grantham (*Science*, 1974, 185, 862-864) [most similar aa are Leu/Ile with D = 5; most distant aa are Cys/Trp with D = 215].

[c] Conservation of aa and location within regions of similarity (Blocks (Bl) I-V) among L proteins of NNS viruses according to [73].

[d] aa change D39F if both G8552 and A8553 changed to U on the same RNA molecule

Abbreviations: s = silent mutation; cs= conservative aa substitution; rs= radical substitution; c= conserved aa in L protein among NNS viruses; nc = non-conserved aa in L protein among NNS viruses; M-GE, F-GE, HN-GE: Gene end (GE) signals of M, F, or HN genes, respectively; M3UTR= 3'UTR region of M gene; N5UTR= 5' UTR region of N gene; Ins= insertion of one nucleotide (A) in homopolymeric tracts of M-GE, F-GE and HN-GE; Bl =block
